# Surveillance of ticks and tick-borne microorganisms in two natural protected areas in Andalusia, South of Spain

**DOI:** 10.1186/s13071-026-07580-1

**Published:** 2026-08-10

**Authors:** Ana M. Palomar, Aránzazu Portillo, Sonia Santibáñez, Cristina San José, Elena Rayas, Ventura Talavera, Eugenio Mallofret, Elena Migens, José A. Oteo

**Affiliations:** 1 Departamento de Enfermedades Infecciosas, Centro de Rickettsiosis y Enfermedades Transmitidas por Artrópodos Vectores (CRETAV), Hospital U. San Pedro-CIBIR, Logroño, Spain; 2 Agencia de Medio Ambiente y Agua de Andalucía, Consejería de Sostenibilidad y Medio Ambiente, Junta de Andalucía, Seville, Spain

**Keywords:** Ticks, Tick-borne bacteria, Tick-borne viruses, Natural protected areas, Doñana, Los Alcornocales, Spain

## Abstract

**Background:**

Spain has two of the most singular ecosystems of Europe with exclusive habitats and unique biodiversity, Doñana and Los Alcornocales, that are key nodes along the East Atlantic flyway between Europe and Africa. The high biodiversity supports a relevant wildlife richness in continuous dispersion that may favor the emergence of tick-borne pathogens, but data from these environments remain scarce.

**Methods:**

From April 2018 to March 2019, questing ticks and associated microorganisms (*Borrelia* spp., spotted fever group *Rickettsia* spp., *Anaplasma* spp., *Ehrlichia* spp., *Neoehrlichia* spp., and Crimean-Congo hemorrhagic fever virus) were monitored.

**Results:**

Questing ticks were found in all the sampling campaigns, and 4726 specimens belonging to 10 species, dominated by *Hyalomma lusitanicum*, were collected. *Candidatus* Rickettsia rioja, *R. slovaca*, *R. raoultii*, *R. aeschlimannii*, *R. monacensis*, *R. barbariae*, *R. massiliae*, *Borrelia lusitaniae*, and *Ehrlichia* species were detected. Viruses were not amplified.

**Conclusions:**

These results highlight the protected areas as natural foci of tick-borne pathogens and underscore the need for integrated surveillance.

**Graphical Abstract:**

**Figure:**
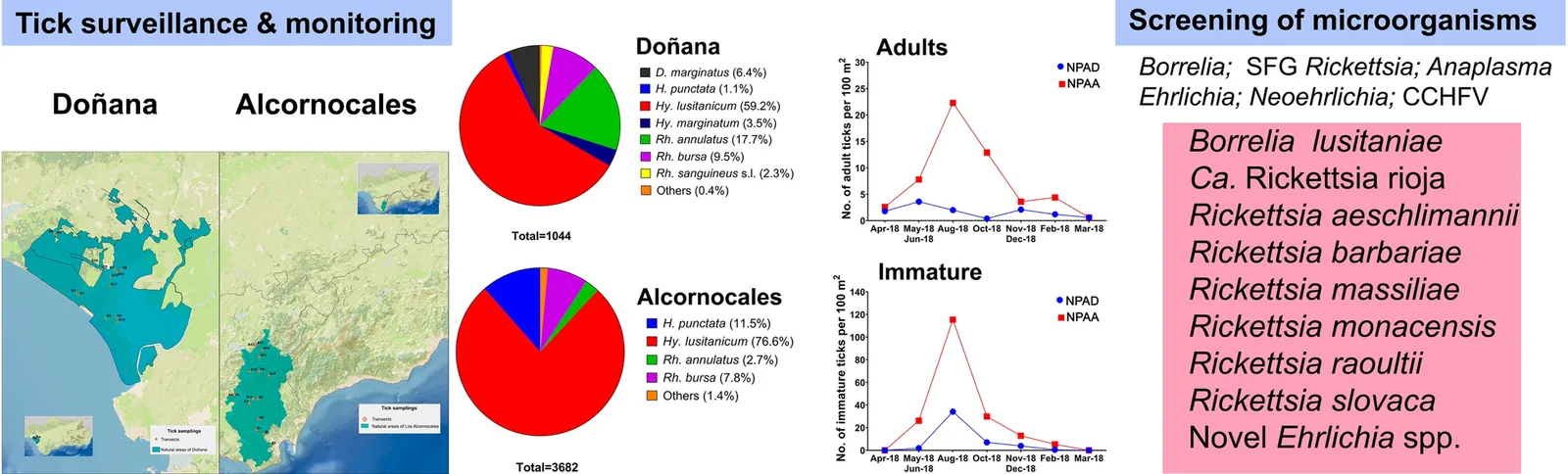

**Supplementary Information:**

The online version contains supplementary material available at https://doi.org/10.1186/s13071-026-07580-1.

## Background

Natural protected areas (NPAs) are geographical spaces managed for the conservation of their ecosystems due to their exceptional value. Their management often focuses on vertebrate species that may act as reservoirs or amplifiers of tick-borne human pathogens and/or dispersers of ticks responsible for zoonosis [1]. NPAs harbor enzootic cycles of tick-borne microorganisms and could be potential hotspots for the occurrence of spillover events into human populations due to the increasing tourism and climatic extreme events joint to the warming weather. Linking tick-borne disease (TBD) emergence and protected areas is complex because it largely depends on the landscape structure, the dynamics of the wildlife communities, and other interconnected factors [2]. Nevertheless, ecotourism is increasing in developed countries, and these areas with recreational services become significant exposure sites for tick-bites among inhabitants, workers, naturalists and visitors. The distribution and abundance of vector species and their associated microorganisms are key factors involved in the occurrence of TBDs [3]. Therefore, monitoring ticks and tick-borne microorganisms is necessary to establish efficient management plans of conservation and to prevent outbreaks of TBDs.

Twenty-three percent of the Spanish territory is included in its NPA network, under different categories (https://www.miteco.gob.es/es/biodiversidad/temas/espacios-protegidos/red-natura-2000/lic.aspx). The NPA of Doñana (hereinafter Doñana), among the most important ones in Europe, and the NPA of Los Alcornocales (hereinafter Alcornocales) are located in the South of the Iberian Peninsula (Andalusia). Both of these NPAs not only support endangered species but also serve as critical corridors and stopovers on the East Atlantic flyway between Europe and Africa. Migratory songbirds are responsible for the spread of ticks and their associated microorganisms, and these migratory routes significantly affect tick-borne pathogen epidemiology [4,5]. Given the singularity of these NPAs, the role of ticks as vectors of pathogens, and the scarce information from the South of Spain, the Agencia de Medio Ambiente y Agua de Andalucía (AMAYA) (Seville, Spain; https://www.juntadeandalucia.es/organismos/amaya.html) and the Centro de Rickettsiosis y Enfermedades Transmitidas por Artrópodos Vectores (CRETAV) (Logroño, Spain; https://www.cibir.es/es/grupos-de-investigacion/enfermedades-infecciosas/artropodos-vectores), with the support of Fundación la Caixa (https://fundacionlacaixa.org/), performed a study of ticks and tick-borne microorganisms from Doñana and Alcornocales. The aim of this work was to investigate the hard tick diversity and abundance, and the presence of *Borrelia* spp., Spotted Fever Group (SFG) *Rickettsia* spp., *Anaplasma* spp., *Ehrlichia* spp. and *Neoehrlichia* spp. in these arthropods. Additionally, a second aim was to investigate the presence of Crimean–Congo hemorrhagic fever virus (CCHFV) in selected samples.

## Methods

### Study areas and tick sampling

**Fig. 1:**
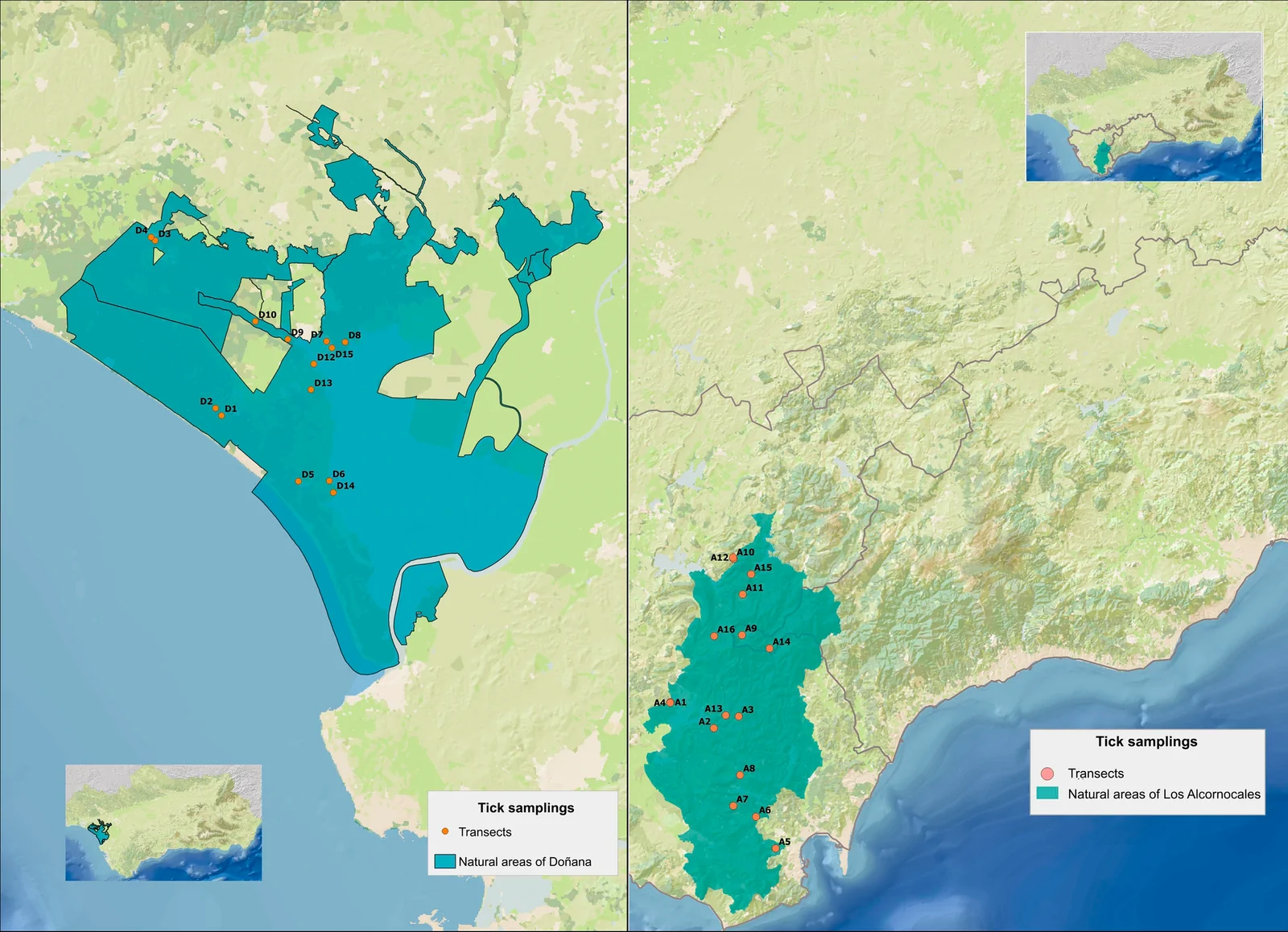
Maps of the study areas showing the transects for tick samplings (Andalusia, southern Spain). Left: Natural Protected Area of Doñana (Huelva), right: Natural Protected Area of Los Alcornocales (Cádiz and Málaga)

Tick sampling was performed in Doñana and Alcornocales (Fig. 1). Both areas have a sub-humid Mediterranean climate with Atlantic influence characterized by hot, dry summers and mild wet winters. Average annual temperatures range from 12 to 25 ºC, and annual variable rainfalls are around 500–600 mm in Doñana and 600–2100 mm in Alcornocales.

Doñana, protected since 1968, is a World Heritage Site and Biosphere Reserve recognized by UNESCO that belongs to the Natura 2000 network (EU). It is the largest wetland in Western Europe (537 km^2^, from the sea level to 47 m.a.s.l.), where marshlands, phreatic lagoons, dunes and Mediterranean terrestrial plant communities are represented and host a significant faunal richness.

Alcornocales, protected since 1989, is the first Intercontinental Biosphere Reserve declared by UNESCO and belongs to the Natura 2000 network. It is one of the largest NPA of Spain (1736 km^2^) dominated by Mediterranean forests with the most southerly cork oak forests (*Quercus suber*) in Europe.

The diversity of ecosystems and conservation efforts rise in an extraordinary ecological richness, making Doñana a critical area for waterfowl and migratory bird species between Africa and Europe, including threatened species such as the marbled teal (*Marmaronetta angustirostris*), the white-headed duck (*Oxyura leucocephala*), the ferruginous duck (*Aythya nyroca*), the bittern (*Botaurus stellaris*) and the black tern (*Chlidonias niger*). Furthermore, Doñana is relevant for supporting other endangered species such as the Iberian lynx (*Lynx pardinus*) and the Imperial eagle (*Aquila adalberti*) (https://whc.unesco.org/en/list/685/). These ecosystems, which promote high faunal density, ensure the maintenance of tick populations.

Sampling areas were selected to cover the main biotopes (pine forest, cork oak forest, Mediterranean shrubs, river valley forests, grassland and marshes), according to the park rangers’ awareness of the tick presence. Questing ticks were searched over 100 m^2^ at 15 sites in Doñana (Huelva) and 16 in Alcornocales (14 sites in Cádiz and two in Málaga) (Fig. 1) by dragging/flagging [6]. Ticks were removed with forceps and kept in tubes according to the area, transect and date.

Seven sampling campaigns were carried out by two collectors about every 2 months from April 2018 to March 2019. In each campaign, 14–15 transects were sampled in Doñana, and 11–14 in Alcornocales. In addition, supplementary sampling was conducted in 100 m^2^ adjacent buffer areas to enhance the sample size (Supplementary Tables 1–2). These buffer areas corresponded to new transects located less than 0.5 km from an ordinary sampling site that shared the same ecological conditions. These areas were punctually sampled following the methodology described above, and the data obtained were considered for the analysis of the tick abundance and associated microorganisms. The air temperature and relative air humidity were measured during fieldwork.

Ticks from campaigns 1–4 were preserved in ethanol 70%; in subsequent campaigns, the specimens were kept alive until their identification and hereafter frozen at  −  80º C without any preservation solution.

Tick abundance was standardized and expressed as the number of individuals collected per 100 m^2^ of sampled area.

### Tick identification, processing and screening of microorganisms

Ticks were identified using morphological keys or descriptions of species [7–10]. Morphological assignments were corroborated and/or complemented by molecular characterization using the 16S rRNA mitochondrial gene (Supplementary Table 3). While *Ixodes inopinatus* is currently considered a synonym of *I. ricinus* [11], we used a previously described multiplex PCR (Supplementary Table 3) to investigate the genotypes present among ticks morphologically identified as *I. ricinus*.

Microbial analysis was performed on all specimens, with the exception of highly abundant species, for which only a selected subsample was processed. The subsample was selected according to the available resources and ensuring that all the species and stages collected during each survey were properly represented. Ticks were individually washed in ethanol 70%, rinsed twice in sterile water and dried (by placing the ventral and dorsal surfaces in contact on a sterile paper tissue and allowing them to air-dry for 2 min) before pooling. Adult ticks were bisected with sterile scalpels for processing. Prior to pooling, a single leg from each *Dermacentor* spp. specimen was dissected for subsequent individual DNA analysis of certain microorganisms. Ticks were grouped according to transect, collection date, tick species, stage and sex. Pools consisted of one to nine adult tick halves or groups of whole immature specimens, containing up to 28 nymphs or 101 larvae per pool. Remaining tick halves and non-tested whole specimens were stored at − 80ºC without adding any preservation solution.

DNA from *Dermacentor* spp. legs was extracted using ammonium hydroxide 0.7 M [12] and analyzed for SFG *Rickettsia* spp. due to the high known prevalence of this bacterial group associated to this tick genus. Its presence in the hemolymph makes legs suitable samples for the *Rickettsia* spp. screening [13].

DNA from pools was extracted using DNeasy Blood & Tissue Kit (Qiagen, Hilden, Germany) and bacteria belonging to species of *Borrelia*, SFG *Rickettsia*, *Anaplasma*, *Ehrlichia* and *Neoehrlichia* were screened using PCR assays (Supplementary Table 3).

RNA extracts from *Hyalomma* ticks (only from those ones preserved at − 80ºC) and reverse-transcribed cDNAs were obtained using RNeasy Mini Kit and Omniscript Reverse transcription kit (Qiagen, Hilden, Germany), respectively, and tested for the presence of CCHFV by PCR (Supplementary Table 3).

Negative controls of extraction, negative controls of PCR (water instead of nucleic acid extracts) and positive controls [DNA or cDNA of *Borrelia spielmanii*, *Borrelia miyamotoi*, *Rickettsia amblyommatis*, *Anaplasma phagocytophilum*, *Ehrlichia* sp. and CCHFV from our ‘Zoonosis collection’, registered in the National Registry of Biobanks from Carlos III Health Institute (Reference: C.0006409) or kindly provided by colleagues, as indicated in the acknowledgments section] were included in all PCR assays.

### Sequence analysis and phylogeny

PCR amplicons of the expected size were sequenced in both senses using the BigDye^®^ Terminator v3.1 Cycle Sequencing Kit (Applied Biosystems, Forest City, CA, USA). Sequencing products were resolved on an ABI PRISM 3130 Genetic Analyzer (Applied Biosystems) at the Molecular Diagnosis Department from CIBIR (Centro de Investigación Biomédica de La Rioja, Logroño, Spain). Nucleotide sequences were analyzed and compared with those available on the NCBI database using the BLAST search tool (http://blast.ncbi.nlm.nih.gov/Blast.cgi). The obtained sequences that differed from those available were submitted through GenBank submission portal (https://submit.ncbi.nlm.nih.gov/).

Phylogenetic analyses were conducted using MEGA 12 (www.megasoftware.net) using the maximum likelihood method and general time reversible model, with a Gamma distribution and invariant sites, including all sites. The nucleotide substitution model was selected according to the Akaike information criterion implemented in MEGA 12. Confidence values for the individual branches were determined by bootstrap analysis with 500 replicates.

### Infectious rates and statistical analysis

Descriptive statistical analyses were presented as frequencies and percentages.

The minimum infection rate (MIR) was calculated, as described elsewhere [14], as the ratio:

$$MIR\, = \,\left( {{{No.\,of\,positive\,pools} \mathord{\left/ {\vphantom {{No.\,of\,positive\,pools} {No.\,\,of\,ticks\,included\,on\,all\,the\,pools\,tested}}} \right. \kern-0pt} {No.\,\,of\,ticks\,included\,on\,all\,the\,pools\,tested}}} \right)\, \times \,100$$

A positive PCR result from a pool was due to the presence of at least one positive specimen, and the MIR assumes that exactly one specimen is infected in each positive pool.

Confidence intervals (95% CI) were calculated using Epitools Epidemiological Calculators (http://epitools.ausvet.com.au) and the Wilson scoring method.

## Results

### Tick species, distribution and abundance

A total of 4726 questing tick specimens (1044 in Doñana and 3682 in Alcornocales) were collected. They belonged to these species: *Dermacentor marginatus*, *Haemaphysalis punctata*, *Hyalomma lusitanicum*, *Hyalomma marginatum*, *Ixodes acuminatus*, *I. ricinus*, *Rhipicephalus annulatus*, *Rhipicephalus bursa*, *Rhipicephalus pusillus* and *Rhipicephalus sanguineus* sensu lato (Table 1). All species were common to both study areas, excluding *Hy. marginatum* and *I. acuminatus*, which were restricted to the Doñana and Alcornocales, respectively. The most prevalent tick species was *Hy. lusitanicum* (*n* = 3438; 72.8%) followed by *H. punctata* (*n* = 434; 9.2%) and *Rh. bursa* (*n* = 385; 8.2%). Within these species, most specimens were immature (79.3%, 88.2% and 77.1% respectively). The highest number of adult ticks in Alcornocales corresponded to *Hy. lusitanicum* (*n* = 647; 80.2%), whereas the most prevalent adult species in the Doñana was *D. marginatus* (*n* = 67; 34.9%) followed by *Hy. lusitanicum* (33.9%). In addition, *Hy. lusitanicum* was the most prevalent tick species in both NPA separately (Doñana = 59.2% and Alcornocales = 76.6%) followed by *Rh. annulatus* in Doñana (*n* = 185, 17.7%, all larvae except one nymph) or by *H. punctata* in Alcornocales (*n* = 423, 11.5%) (Supplementary Figs. 1–2).

**Table 1:** Ticks collected in the Natural Protected Areas of Doñana and Los Alcornocales and studied from April 2018 to March 2019

Origin	Tick species	No. of ticks collected/no. of ticks processed for the screening of microorganisms (no. of pools)				
		Females	Males	Nymphs	Larvae	Total
Natural Protected Area of Doñana	*Dermacentor marginatus*	37/37 (9)	30/30(9)			67/67 (18)
	*Haemaphysalis punctata*	2/2 (1)		9/9 (1)		11/11 (2)
	*Hyalomma lusitanicum*	32/32 (7)	33/33(6)	182/93 (6)	371/200 (2)	618/358 (21)
	*Hyalomma marginatum*	1/1 (1)			35/35 (1)	36/36 (2)
	*Ixodes ricinus*		2/2 (1)			2/2 (1)
	*Rhipicephalus annulatus*			1/1 (1)	184/184 (4)	185/185 (5)
	*Rhipicephalus bursa*	8/8 (2)	21/16(3)		70/70 (1)	99/94 (6)
	*Rhipicephalus pusillus*	1/1 (1)	1/1(1)			2/2 (2)
	*Rhipicephalus sanguineus* sensu lato	12/12 (4)	12/12(4)			24/24 (8)
Natural Protected Area of Los Alcornocales	*D. marginatus*	3/3 (3)	1/1 (1)			4/4 (4)
	*H. punctata*	18/18 (4)	31/31 (6)	55/55 (5)	319/319 (5)	423/423 (20)
	*Hy. lusitanicum*	304/212(29)	343/243 (34)	181/166 (8)	1992/791 (9)	2820/1412 (80)
	*Ixodes acuminatus*		1/1 (1)			1/1 (1)
	*I. ricinus*	2/2 (2)	3/3 (3)	1/1 (1)		6/6 (6)
	*Rh. annulatus*				101/101 (1)	101/101 (1)
	*Rh. bursa*	30/20 (4)	29/19 (3)		227/227 (5)	286/266 (12)
	*Rh. pusillus*	7/7 (3)	2/2 (2)			9/9 (5)
	*Rh. sanguineus* s.l.	18/18 (5)	14/14 (3)			32/32 (8)

Tick abundance ranged from 0.6 to 36.2 and from 0.6 to 137.8 tick specimens per 100 m^2^ in Doñana and Alcornocales, respectively (Table 2, Supplementary Fig. 3). The highest density was found in August 2018 in both areas, corresponding with the largest captures of immature individuals (Supplementary Figs. 3–5). Tick abundance was higher in Alcornocales than in Doñana for all sampling campaigns.

**Table 2:** Tick abundance, calculated as tick number per 100 m^2^ sampled, in the study areas of Doñana and Alcornocales from April 2018 to March 2019

Sampling campaign (date)	Doñana	Alcornocales
S-1 (Apr-18)	1.8	2.6
S-2 (May-18/Jun-18)	5.5	33.9
S-3 (Aug-18)	36.2	137.8
S-4 (Oct-18)	7.5	42.7
S-5 (Nov-18/Dec-18)	5.9	16.5
S-6 (Feb-19)	1.6	9.8
S-7 (Mar-19)	0.6	0.6

The number of ticks, species and stage per transect and per sampling campaign are detailed in Supplementary Tables 4–11. The tick densities reached up to 51.5 specimens per 100 m^2^, in the FD8 buffer area from Doñana and 378.2 specimens per 100 m^2^ in transect A16 from Alcornocales (Supplementary Table 12). All developmental stages of *Hy. lusitanicum* were recovered from these transects. For the transects sampled across all campaigns, the highest densities reached 31.6 (transect D8) and 37.1 (transect A13) ticks per 100 m^2^ in Doñana and Alcornocales, respectively.

According to the seasonality, different patterns of abundance were obtained depending on the tick species. *Hy. lusitanicum*, reached its peak abundance in summer, with the presence of all life stages. Densities in spring and autumn remained notable (Supplementary Fig. 6). The tick distribution of other prevalent tick species is represented in Supplementary Figs. 7–10. *H. punctata* larvae were mainly found in spring, while adults and nymphs of this species were collected at the end of winter. As expected, *D. marginatus* occurred mainly in cold months (November to February). Adults of *Rh. bursa* were mainly found in summer and larvae, in winter. Immature of *Rh. annulatus* were abundant in autumn. The temperatures and relative humidity reached during surveys was recorded in Supplementary Tables 13 and 14.

The morphological classification was corroborated by genetic identification in four *H. punctata*, 29 *Hy. lusitanicum* (mainly immature specimens), three *Hy. marginatum*, one *I. acuminatus*, two *I. ricinus*, four *Rh. annulatus*, two *Rh. bursa*, one *Rh. pusillus* and four *Rh. sanguineus* s.l. Percentages of identity with available sequences are shown in Supplementary Table 15. Moreover, multiplex PCR for ticks morphologically identified as *I. ricinus* yielded a single band corresponding to *I. inopinatus* in all (*n* = 6) but one sample, which showed two bands, suggesting a potential hybrid. Nevertheless, and given that further investigation is needed on the taxonomic status *I. inopinatus*, currently considered a synonym of *I. ricinus* [11]; herein, we will refer to these specimens as *I. ricinus*.

### Tick-borne microorganisms

A total of 3033 ticks grouped in 202 pools were selected for microorganisms screening (Table 3). CCHFV screening was performed in 16 pools of *Hy. lusitanicum*, three of them (five specimens) from Doñana and 13 pools (74 specimens) from Alcornocales.

**Table 3:** Microorganisms detected in ticks from the Natural Protected Areas of Doñana and Los Alcornocales from April 2018 to March 2019

Origin	Tick species	No. of ticks analyzed/no. of pools	Microorganisms; no. of positive pools (no. of each stage)/MIR % (CI)		
			*Borrelia* spp.	*Rickettsia* spp.	Anaplasmataceae
Natural Protected Area of Doñana	*Dermacentor marginatus*	67/18		*Candidatus* Rickettsia rioja*; 40 (24 F, 16 M)/59.7 (47.7–70.6) Rickettsia slovaca*; 21 (12 F, 9 M)/31.3 (21.5–43.2)	
	*Haemaphysalis punctata*	11/2			
	*Hyalomma lusitanicum*	358/21			
	*Hyalomma marginatum*	36/2		*Rickettsia aeschlimannii;* 2 (1 F, 1 L)/5.6 (1.5–18.1)	
	*Ixodes ricinus*	2/1	*Borrelia lusitaniae*; 1(1 M)/50 (9.5–90.6)	*Rickettsia monacensis;* 1 (1 M)/50 (9.5–90.6)	
	*Rhipicephalus annulatus*	185/5			
	*Rhipicephalus bursa*	94/6		*Rickettsia barbariae;* 1 (1 M)/1.1 (0.2–5.8)	
	*Rhipicephalus pusillus*	2/2			
	*Rhipicephalus sanguineus* s.l	24/8		*Rickettsia massiliae;* 8 (4 F, 4 M)/33.3 (18.0–53.3)	
Natural Protected Area of Los Alcornocales	*D. marginatus*	4/4		*Ca.* R. rioja*; 3 (3 F)/75 (30.1–95.4) Rickettsia raoultii*; 1 (1 M)/25 (4.6–70.0)	
	*H. punctata*	423/20			
	*Hy. lusitanicum*	1412/80			*Ehrlichia* spp.; 7 (2 F, 1 M, 4 N)/0.5 (0.2–1.0)
	*Ixodes acuminatus*	1/1			
	*I. ricinus*	6/6	B. lusitaniae*; 5 (2 F, 3 M)/83.3 (43.7–97.0)	R. monacensis*; 4 (2 F, 1 M, 1 N)/66.7 (30.0–90.3)	
	*Rh. annulatus*	101/1			
	*Rh. bursa*	266/12		*R. massiliae;* 1(1 M)/0.4 (0.001–2.1) *R. barbariae*; 3 (1 F, 1 M, 1 L)/1.1 (0.4–3.3)	
	*Rh. pusillus*	9/5			
	*Rh. sanguineus* s.l	32/8		*R. massiliae;* 7 (5 F, 2 M)/21.9 (11.2–38.8)	
Total		3033/202			

*s.l.* sensu lato, *MIR* Minimum infectious rate, *CI* 95% Confidence Interval, *F* Female, *M* Male, *N* Nymph, *L* Larvae

*Detected in individual tick specimens, MIR should be considered as prevalence

The microorganisms detected are summarized in Table 3, according to the sequence analysis (Supplementary Table 15).

*Borrelia lusitaniae* was amplified in the single pool with two *I. ricinus* specimens from Doñana (MIR = 50%; CI 9.5–90.6%), and in 5/6 specimens of *I. ricinus* from Alcornocales (prevalence of 83.3%; CI 43.7–97%).

Pools of *I. ricinus* were also infected with *Rickettsia monacensis* (MIR = 62.5%), specifically, the pool of Doñana (MIR = 50%; CI 9.5–90.6%), and 4/6 specimens collected in Alcornocales (66.7% of prevalence; CI 30.0–90.3%). Other *Rickettsia* spp. amplified were *Rickettsia aeschlimannii*, in the two *Hy. marginatum* pools from Doñana (MIR = 5.6%; CI 1.5–18.1%); *Rickettsia massiliae*, in all but one *Rh. sanguineus* s.l. pools (MIR = 26.8%; CI 18.0–39.6%), eight from Doñana and seven from Alcornocales, and in a single pool of *Rh. bursa* (MIR = 0.3%; CI 0.001–1.6%) from Alcornocales; *Rickettsia barbariae*, 1/6 pools and 3/12 *Rh. bursa* pools (MIR = 1.1%; CI 0.4–2.8%) from Doñana and Alcornocales, respectively; and *Candidatus* Rickettsia rioja, *R. slovaca* or *R. raoultii* in 61 and four of the *D. marginatus* specimens studied from Doñana (91% of prevalence; CI 81.8–95.8%) and Alcornocales (100% of prevalence; CI 51.1–100%), respectively. Specifically, 43 of these amplicons corresponded to *Ca*. R. rioja (60.6%; CI 48.9–71.1%), 21 to *R. slovaca* (29.6%; CI 20.2–41.0%) and one to *R. raoultii* (1.4%; CI 0.3–7.6%).

**Fig. 2:**
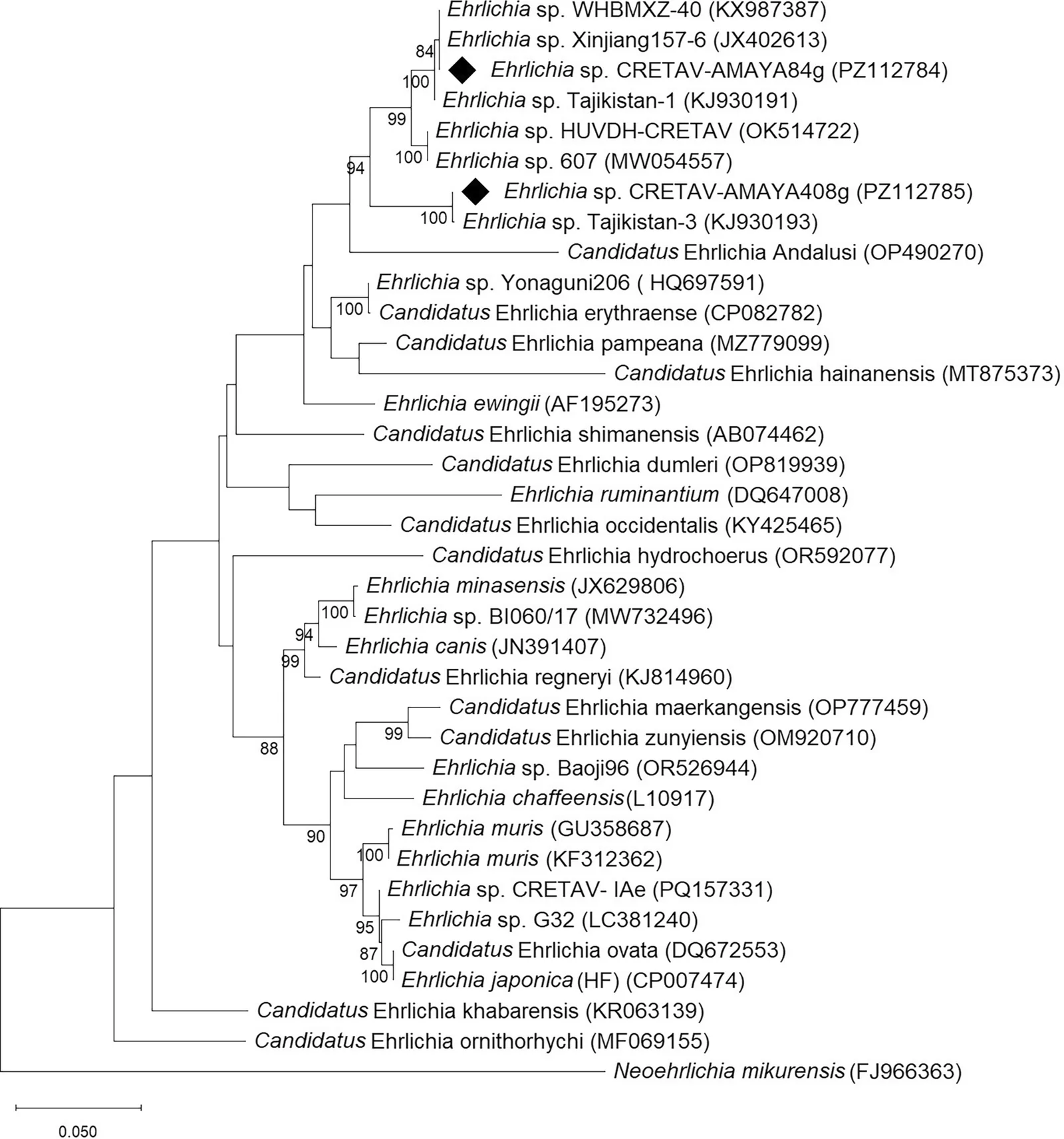
Phylogeny of *Ehrlichia* spp. inferred with *gro*ELS sequences using the maximum likelihood method and general time reversible model (500 replicates) with a discrete gamma distribution and sites deemed evolutionarily invariant, showing the position of the *Ehrlichia* spp. amplified in the present study (◆). This analysis involved 36 nucleotide sequences with 1066 positions in the final dataset with *Neoehrlichia mikurensis* as outgroup. Replicate numbers are shown in the nodes

In seven out of 101 *Hy. lusitanicum* pools (MIR = 0.4%; CI 0.2–0.8%) from Alcornocales, *Ehrlichia* spp. were detected with partial *gro*ESL, 16S rRNA and *gltA* PCR assays. Two different genotypes showed < 94% identity with *gro*ESL of *Ehrlichia ewingii* as the nearest validated species (GenBank accession No. AF195273). The highest identities (99.9–100%) were reached with unclassified *Ehrlichia* spp. found in *Hyalomma* spp. or *Rhipicephalus microplus* from Asia (e.g., OQ185227 or KJ930193) (Fig. 2).

GenBank accession numbers of sequences obtained in this study are shown in Supplementary Table 15.

Relapsing fever group *Borrelia* spp., *Anaplasma* spp., *Neoehrlichia* spp., or CCHFV were not detected with the PCR targets used.

## Discussion

In this study, the diversity and abundance of questing tick species and their associated microorganisms were investigated in Doñana and Alcornocales (southern Spain). Ten different tick species and bacteria belonging to three genera, some of them associated with human diseases, were found.

Sampling sites were selected to ensure the tick monitoring in the different habitats of both study areas, but in some cases, priority was given to areas where ticks were known to be present, based on prior knowledge of their presence. Therefore, it is likely that data from these few areas slightly overestimated tick abundance, but did not affect the composition of tick species, which is primarily biased by the sampling method, and may have improved the screening of microorganisms, which is often biased mainly due to the small sample size.

The occurrence of all sampled tick species in this study was expected since they had been previously recorded in Andalusia, southern Spain [10, 15,16]. Nevertheless, it is important to recognize that species, stages and abundance may be biased by the selected sampling method [17]. Thus, our sampling technique is optimal for collecting questing species or stages, such as *I. ricinus*, but maybe less effective for those ticks with active host-seeking, nidicolous or with on-host molting. This fact could explain the absence of certain tick species such as *I. hexagonus*, immature *D. marginatus* specimens, adult *Rh. annulatus* individuals, or the low density of *I. acuminatus*, *Hy. marginatum* or *Rh. pusillus*.

Given that the risk of human exposure to tick-bites is strongly associated with questing tick abundance [18], our data demonstrate a year-round risk of being bitten in both NPAs, and particularly during the summer in both areas, despite the fact that overall tick densities were higher in Alcornocales than in Doñana. According to our data, we are not able to relate the tick density with type of habitats because the abundance of ticks in transects with similar habitats were highly variable (i.e., D8 and D3 or A7 and A8) and close densities were found in transects with different habitats (i.e. D8 and D13 or A6 and A14). Further studies considering aspects such as hosts, habitats of neighboring areas, etc. should be performed.

Regarding the detected microorganisms, it is worth mentioning that pooled samples were screened and the prevalence rates were calculated assuming that only one specimen per pool was positive (MIR). This is a downward estimation. It is therefore likely that the prevalence rates showed herein are underestimated, particularly in large pools.

*Borrelia* sp. was amplified in *I. ricinus* specimens, and *B. lusitaniae* was the unique species detected. Despite the low number of analyzed ticks, the MIR reached 75% (CI 40.9–92.9%), a percentage similar to those found in areas of Portugal [19]. Lower infectious rates of *B. lusitaniae* have been reported in the vector not only in the Mediterranean basin but also in other regions (e.g., northern Spain and Central Europe) where other *Borrelia* spp. occur, including recognized human pathogenic species of *B. burgdorferi* sensu lato complex and/or *B. miyamotoi* [12, 20,21]. The pathogenicity of *B. lusitaniae* is controversial. It has been detected or isolated from human samples but there are scarce cases, with absence of serological evidence and non-specific clinical symptoms [20, 22–25]. Our findings, particularly the absence of *B. burgdorferi* s.l. genospecies clearly associated with human disease, may explain the limited reporting or absence of Lyme borreliosis in the southern Spain. This is also reinforced by a very low abundance of *I. ricinus*, a very limited circulation of *B. burgdorferi* s.l. genospecies recognized as relevant cause of human disease and very infrequent clinical cases of Lyme borreliosis clearly documented, according to studies from the South of Spain [26].

Herein, seven SFG *Rickettsia* species were detected in questing ticks. Among the human pathogens identified, *Ca.* R. rioja, *R. slovaca* and *R. raoultii* were those with the greatest impact on human health since they are the etiological agents of *Dermacentor*-borne-necrosis-erythema-lymphadenopathy/tick-borne-lymphadenophathy (DEBONEL/TIBOLA) [13]. High prevalence of these rickettsiae was observed in *D. marginatus*, the competent vector, in agreement with previous reports from the study area [27]. Specifically, *Ca*. R. rioja emerged as the most prevalent species, as previously observed in ticks from patients included in the largest reported series of DEBONEL [13]. Moreover, three additional *Rickettsia* species (*R. monacensis*, *R. aeschlimannii* and *R. massiliae*) associated with human zoonotic diseases were found. The presence of *R. monacensis*, previously known as “agent Cádiz”, had already been reported in the area [15]. This species is distributed across North Africa, Europe and Asia and *Ixodes* spp. (*I. ricinus* in Europe) are suspected vectors. In this study, the infection rate in *I. ricinus* was higher than those previously found in ticks from animals in Andalusia [16, 28]; however, our results could be biased by the few analyzed specimens. Despite its high prevalence and wide distribution, only a few human cases caused by *R. monacensis* have been described in Europe and Asia [29–31]. *R. aeschlimannii* was amplified in the two *Hy. marginatum* pools analyzed, which is the suspected vector in Europe. The pathogenic role of *R. aeschlimannii* seems to be low, despite its high prevalence rate in the vector [32,33]. Nevertheless, human cases from Asia have been recently added to those reported from North Africa and Europe [30, 34–36]. The presence of *R. massiliae* in the study area had been previously demonstrated [28], and prevalence rates ranging from 2 to 92% have been documented in European ticks [37]. *R. massiliae* human infections have been punctually reported from different continents, including one case from Spain acquired in Argentina [30, 38–40]. Lastly, this study corroborates the circulation of *R. barbariae* in the Iberian Peninsula, since it was initially detected in *Rh. bursa* ticks in Portugal and provisionally named *Rickettsia* sp. PoTiRb169 [41]. To date, *R. barbariae* has not been involved in human health, but molecular evidence of its presence in anthropophilic ticks (mainly *Rhipicephalus* spp.) has been reported from Europe, Africa and Asia [41–43].

Two putative novel *Ehrlichia* genotypes not previously reported in Spain have been amplified in *Hy. lusitanicum* from Alcornocales. According to the fragment genes analyzed, they belong to unclassified *Ehrlichia* spp., mainly found in Asia [44–46]. Potential novel *Ehrlichia* species have been partially characterized worldwide, including those from Spain [14, 47,48]. A more comprehensive characterization of these genotypes, including isolation, spatial distribution, and identification of their vectors and reservoirs, is urgently required to better understand and prevent emerging TBDs. Thus, unclassified *Ehrlichia* species such as *Candidatus* Ehrlichia erythraense has been recently involved as causative agents of a febrile illness outbreak in China [49].

In our study, CCHFV was not detected, although these results should be interpreted with caution due to the limited number of specimens analyzed. Moreover, the absence of viral detection does not exclude local circulation because the circulation of the virus in Spain and the emergence of endemic cases of the disease is known for at least 2 decades, and the virus has been recently described in the study area [50–52]. Furthermore, new tick-borne viruses are being described in Spain [14]. These facts make necessary to carry out new tick-borne virus surveillance programs in this area.

## Conclusions

The presence of ticks that are recognized vectors of human diseases and the circulation of tick-borne zoonotic agents in Doñana and Alcornocales (South of Spain) highlight the potential public health relevance of tick exposure in these areas, which in turn could favor zoonotic spillover events. Our results emphasize the need for continuous molecular surveillance of tick populations in these strategic areas, promotion of preventive measures to reduce human-tick contacts and mitigate infection risks, and enhanced clinical awareness to improve the diagnosis of tick-borne infections.

## Supplementary Information

Supplementary Material 1.

Supplementary Material 2.

## Data Availability

All the data are available in the main text or in the supplementary material. The obtained sequences were deposited in the GenBank database under the accession numbers shown in the fifth column of Supplementary Table 15.
