# Supplementary material for: Surveillance of ticks and tick-borne microorganisms in two natural protected areas in Andalusia, South of Spain
Source: Parasit Vectors. 2026 Aug 10;19:476. doi: 10.1186/s13071-026-07580-1 (PMC13737913; doi:10.1186/s13071-026-07580-1)
Supplement: Supplementary file 1 [file 13071_2026_7580_MOESM1_ESM.pdf]

Supplementary table 1. Transects where tick samplings were performed in the Natural Protected Area of Doñana. Dates in which each transect was sampled is marked in grey.

Transect*	Province	Municipal boundary	Area	Coordinate		Habitat	Sampling dates						
				**X**	**Y**		**S-1 Apr-18**	**S-2 May-18**	**S-3 Aug-18**	**S-4 Oct-18**	**S-5 Nov-18**	**S-6 Feb-19**	**S-7 Mar-19**
D1	Huelva	Almonte	Laguna de las pajas	181187	4106049	Grassland on the shore of the lake							
D2	Huelva	Almonte	Laguna huerto Bejarano	180510	4106893	The shore of the lake with Mediterranean shrub (rockrose, rosemary, lentisk, etc.)							
D3	Huelva	Bonares	Arboreto del Villar I	173551	4126174	Cork oak and Mediterranean shrub (rockrose, rosemary, lentisk, etc.)							
D4	Huelva	Bonares	Arboreto del Villar II	173085	4126562	Cork oak and Mediterranean shrub (rockrose, rosemary, lentisk, etc.)							
D5	Huelva	Almonte	Laguna de Santa Olalla	190034	4098488	Grassland on the shore of the lake and scrubs of sage-leaved rockrose							
D6	Huelva	Almonte	El Puntal-Helechal	193599	4098535	Fern under a cork oak							
D7	Huelva	Hinojos	Casa del Pichiricha (Coto del Rey)	193272	4114582	Grassland							
D8	Huelva	Hinojos	Raya de los Álamos	195412	4114499	Cork oak and Mediterranean shrub (rockrose, rosemary, lentisk, etc.)							
D9	Huelva	Almonte	Rocina Laguna	188825	4114833	Pine forest on the shore of the lake with fens and scrubs							
D10	Huelva	Almonte	Sendero Acebrón	185082	4116876	Cork oak, Mediterranean shrub and grassland with fan palms							
D11	Huelva	Almonte	Toril de Manecorro	190510	4113701	Grassland close to the lake							
D12	Huelva	Almonte	Casa Hato Villa	191804	4111984	Grassland at the edge of marshes							
D13	Huelva	Almonte	El Guaperal zona de acampada	191484	4109023	Grassland, scrub and eucalyptus							
D14	Huelva	Almonte	Toril Casa del Puntal	194058	4097193	Grassland							
D15	Huelva	Hinojos	Cuevas de Conejos	193897	4113834	Cork oak forest and Mediterranean shrub							

* Neighbouring areas (buffer areas) were sampled in August (n=3; D4, D6 and D8), October (n=9; D1-2, D4, D6-9 and D12-13), November (n=4; D4-7), February (n=3; D5, D7 and D10) and March (n=5; D3-D7).

Supplementary table 2. Transects where tick samplings were performed in the Natural Protected Area of Los Alcornocales (South Spain). Dates in which each transect was sampled is marked in grey.

Transect*	Province	Municipal boundary	Area	Coordinate		Habitat	Sampling dates						
				X	Y		S-1 Apr-18	S-2 Jun-18	S-3 Aug-18	S-4 Oct-18	S-5 Dec-18	S-6 Feb-19	S-7 Mar-19
A1	Cádiz	Alcalá de los Gazules	Cañadas de los ratones. Conejeras	254254	4032969	Grassland	■	■	■	■	■	■	■
A2	Cádiz	Alcalá de los Gazules	La Peguera	262887	4027813	Grassland at the edge of the Mediterranean scrub	■	■	■	■	■	■	■
A3	Cádiz	Alcalá de los Gazules	Finca Buenas Noches	267824	4030159	Grassland at the edge of the Mediterranean scrub (rockrose, rosemary, lentisk, etc.)	■						
A4	Cádiz	Alcalá de los Gazules	Pradera en la Cañana de los ratones	254227	4032857	Grassland	■	■					
A5	Cádiz	Los Barrios	Pantano Monte La Torre	275161	4003948	Grassland on the shore of the lake	■	■	■	■	■	■	■
A6	Cádiz	Los Barrios	Cañada Real Botafuegos-Jaramillo	271244	4010225	Grassland at the edge of the Mediterranean scrub (rockrose, rosemary, lentisk, etc.)	■	■	■	■	■	■	■
A7	Cádiz	Los Barrios	Sendero de Valdeinfierno	266752	4012397	Cork oak forest and Mediterranean scrub (rockrose, heather, etc.)	■	■	■	■	■	■	■
A8	Cádiz	Los Barrios	La Teja	268097	4018499	Grassland and Mediterranean scrub	■	■	■	■	■	■	■
A9	Málaga	Cortes de la Frontera	Cerca de PerezGil	268478	4046307	"Bujeo" grasslands	■	■	■	■	■	■	■
A10	Cádiz	Algar	Carril Finca la Atalaya	266809	4061435	"Bujeo" grasslands next to riverside	■						
A11	Cádiz	Jerez de la Frontera	Arroyo entrada Montes de Jerez	268611	4054374	Riverside meadows	■	■	■	■	■	■	■
A12	Cádiz	Algar	Garganta Peñalpargate	266687	4061715	"Bujeo" grasslands next to riverside	■	■					
A13	Cádiz	Alcalá de los Gazules	Praderas con casas subiendo a Buenas Noches	265239	4030360	Grassland and cork oak forest	■	■	■	■	■	■	■
A14	Málaga	Cortes de la Frontera	Patio de Corcha	273954	4043661	Grassland at the edge of the Mediterranean scrub	■	■	■	■	■	■	■
A15	Cádiz	Jerez de la Frontera	Finca Rojitan, Embalse de los Hurones	270293	4058386	River valley forests				■	■	■	■
A16	Cádiz	Alcalá de los Gazules	CR Corzo, el Picacho	262942	4046108	Cork oak forest and Mediterranean scrub (rockrose, heather, etc.)			■	■	■	■	■

*Neighbouring areas (buffer areas) were sampled in August (n=5; A5-6, A8-9 and A14), October (n=3; A9, A11 and A14), December (n=3; A9, A11 and A13) and February (n=4; A5-7 and A13).

Supplementary table 3: PCR primer pairs and conditions used in this study for the screening of tick-borne microorganisms.

Organisms	Target gene	Primer sequence (5'→ 3')	Fragment size (bp)	References
**Ticks**	16S rRNA	F:CTGCTCAATGATTTTTTAAATTGCTGTGG<br>R:CCGGTCTGAACTCAGATCAAGT	456	[1]
	TROSPA	F: GTAAACATCGGCCTAATGG<br>R: GGAAAAAATAATGTTAAAACACC<br>F: GTTGTTCACAGCGAATACT<br>R: GAAAAAAATATTAGAACATTAACACTC	362*<br>233†	[2]
***Borrelia* spp.**	*flaB* (Lyme group)	F1: AARGAATTGGCAGTTCAATC<br>R1: GCATTTTCWATTTTAGCAAGTGATG<br>F2: ACATATTCAGATGCAGACAGAGGTTCTA<br>R2: GAAGGTGCTGTAGCAGGTGCTGGCTGT	497<br><br>389	[3]<br>[4]
	glpQ (Relapsing fever group)	F1: ATGGGTTCAAACAAAAAGTCACC<br>R1: CATTACTGTGTCAGTAAAATCTGTAAATATACCATCTAC<br>F2: ATGGGTTCAAACAAAAAGTCACC<br>R2: CCAGGGTCCAATTCCATCAGAATATTGTGCAAC	920<br><br>700	[5]<br>[6]
***Rickettsia* spp.**	*ompA* (Spotted fever group)	F1: ATGGCGAATATTTCTCCAAAA<br>R1: GTTCCGTTAATGGCAGCATCT<br>F2: ATGGCGAATATTTCTCCAAAA<br>R2: AGTGCAGCATTCGCTCCCCCT	631<br><br>532	[7,8]
	ompB‡	F1: GTAACCGGAAGTAATCGTTTCGTAA<br>R1: GCTTTATAACCAGCTAAACCACC<br>F2: GTTTAATACGTGCTGCTAACCAA<br>R2: GGTTTGGCCCATATACCATAAG	420<br><br>230	[9]
***Anaplasma /Ehrlichia/ Neoehrlichia* spp.**	groESL	F1: AITGGGCTGGTAITGAAAT<br>R1: CCICCIGGIACIAIACCTTC<br>F2: ATWGCWAARGAAGCATAGTC<br>R2: CTCAACAGCAGCTCTAGTAGC	 1350<br><br>1297	[10]
Anaplasma phagocytophilum	msp2	F1:CCAGCGTTTAGCAAGATAAGAG<br>R1:GCCCAGTAACAACATCATAAGC	380	[11]
***Ehrlichia* spp.** ‡	16S rRNA	F1: GAACGAACGCTGGCGGCAAGC<br>R1: AGTAYCGRACCAGATAGCCGC	696	[12,13]
	gltA	F1:ATGTCTACTGCTGCTTGTGA<br>R1: ATGACCAGTATATAACTGACGTG<br>F2:ATGTCTACTGCTGCTTGTGA<br>R2: GAGCAGACCAACCAGATGTTC	 1098<br><br>1030	[13,14]
**Crimean-Congo haemorrhgic fever virus**‡	S segment	F1: TCTCAAAGAAACACGTGCC<br>R1: CCTTTTTGAACTCTTCAAACC<br>Probe: ACTCAAGGKAACACTGTGGGCGTAAG	122	[15]
		F1: TTGTGTTCCAGATGGCCAGC<br>R1: CTTAAGGCTGCCGTGTTTGC<br>F2: GAAGCAACCAARTTCTGTGC<br>R2: AAACCTATGTCCTTCCTCC	 307<br><br>221	[16]
		F1: AATGTGCCGAACTTGGACAG<br>R1: TGCGACAAGTGCAATCCCG<br>F2: ATCAGATGGCCAGTGCAACC<br>R2: ACTCCCTGCACCACTCAATG	 593<br><br>469	[16]
		F1: AARGGAAATGGACTTRTGGA<br>R1: GCAAGGCCTGTWGCRACAAGTGC<br>F2: TGGAYACYTTCACAAACTCC<br>R2: GCAAGGCCTGTWGCRACAAGTGC	 605<br><br>621	[17]

bp: base pairs; * amplicon size corresponding to *Ixodes ricinus*; †amplicon size corresponding to *Ixodes inopinatus*; ‡Only performed to selected samples

References:

(1) Black WC, Piesman J. Phylogeny of hard- and soft-tick taxa (Acari: Ixodida) based on mitochondrial 16S rDNA sequences. Proc Natl Acad Sci U S A. 1994;91(21):10034-8. doi: 10.1073/pnas.91.21.10034

(2) Hrazdilova K, Danek O, Hrbatova A, Cervena B, Noskova E, Adamik P, Votypka J, Mihalca AD, Noureddine M, Modry D, Zurek L. Genetic analysis challenges the presence of *Ixodes inopinatus* in Central Europe: development of a multiplex PCR to distinguish *I. inopinatus* from *I. ricinus*. Parasit Vectors. 2023;16(1):354. doi: 10.1186/s13071-023-05971-2.

(3) Clark K, Hendricks A, Burge D. Molecular identification and analysis of *Borrelia burgdorferi* sensu lato in lizards in the southeastern United States. Appl Environ Microbiol. 2005;71(5):2616-25. doi: 10.1128/AEM.71.5.2616-2625.2005.

(4) Johnson BJ, Happ CM, Mayer LW, Piesman J. Detection of *Borrelia burgdorferi* in ticks by species-specific amplification of the flagellin gene. Am J Trop Med Hyg. 1992;47(6):730-41. doi: 10.4269/ajtmh.1992.47.730.

(5) Hovius JW, de Wever B, Sohne M, Brouwer MC, Coumou J, Wagemakers A, Oei A, Knol H, Narasimhan S, Hodiamont CJ, Jahfari S, Pals ST, Horlings HM, Fikrig E, Sprong H, van Oers MH. A case of meningoencephalitis by the relapsing fever spirochaete *Borrelia miyamotoi* in Europe. Lancet. 2013;382(9892):658. doi: 10.1016/S0140-6736(13)61644-X.

(6) Wagemakers A, Jahfari S, de Wever B, Spanjaard L, Starink MV, de Vries HJC, Sprong H, Hovius JW. *Borrelia miyamotoi* in vectors and hosts in The Netherlands. Ticks Tick Borne Dis. 2017;8(3):370-374. doi: 10.1016/j.ttbdis.2016.12.012.

(7) Regnery RL, Spruill CL, Plikaytis BD. Genotypic identification of rickettsiae and estimation of intraspecies sequence divergence for portions of two rickettsial genes. J Bacteriol. 1991;173(5):1576-89. doi: 10.1128/jb.173.5.1576-1589.1991.

(8) Roux V, Fournier PE, Raoult D. Differentiation of spotted fever group rickettsiae by sequencing and analysis of restriction fragment length polymorphism of PCR-amplified DNA of the gene

encoding the protein rOmpA. J Clin Microbiol. 1996;34(9):2058-65. doi: 10.1128/jcm.34.9.2058-2065.1996.

(9) Choi YJ, Jang WJ, Kim JH, Ryu JS, Lee SH, Park KH, Paik HS, Koh YS, Choi MS, Kim IS. Spotted fever group and typhus group rickettsioses in humans, South Korea. Emerg Infect Dis. 2005;11(2):237-244. doi: 10.3201/eid1102.040603.

(10) Liz JS, Sumner JW, Pfister K, Brossard M. PCR detection and serological evidence of granulocytic ehrlichial infection in roe deer (*Capreolus capreolus*) and chamois (*Rupicapra rupicapra*). J Clin Microbiol. 2002 Mar;40(3):892-7. doi: 10.1128/JCM.40.3.892-897.2002.

(11) Zeidner NS, Burkot TR, Massung R, Nicholson WL, Dolan MC, Rutherford JS, Biggerstaff BJ, Maupin GO. Transmission of the agent of human granulocytic ehrlichiosis by *Ixodes spinipalpis* ticks: evidence of an enzootic cycle of dual infection with *Borrelia burgdorferi* in Northern Colorado. J Infect Dis. 2000;182(2):616-9. doi: 10.1086/315715.

(12) Rar VA, Livanova NN, Panov VV, Doroschenko EK, Pukhovskaya NM, Vysochina NP, Ivanov LI. Genetic diversity of *Anaplasma* and *Ehrlichia* in the Asian part of Russia. Ticks Tick Borne Dis. 2010;1(1):57-65. doi: 10.1016/j.ttbdis.2010.01.002.

(13) Rar VA, Pukhovskaya NM, Ryabchikova EI, Vysochina NP, Bakhmetyeva SV, Zdanovskaia NI, Ivanov LI, Tikunova NV. Molecular-genetic and ultrastructural characteristics of '*Candidatus* Ehrlichia khabarensis', a new member of the *Ehrlichia* genus. Ticks Tick Borne Dis. 2015;6(5):658-67. doi: 10.1016/j.ttbdis.2015.05.012.

(14) Cruz AC, Zweygarth E, Ribeiro MF, da Silveira JA, de la Fuente J, Grubhoffer L, Valdés JJ, Passos LM. New species of *Ehrlichia* isolated from *Rhipicephalus* (*Boophilus*) *microplus* shows an ortholog of the *E. canis* major immunogenic glycoprotein gp36 with a new sequence of tandem repeats. Parasit Vectors. 2012;5:291. doi: 10.1186/1756-3305-5-291.

(15) Atkinson B, Chamberlain J, Logue CH, Cook N, Bruce C, Dowall SD, Hewson R. Development of a real-time RT-PCR assay for the detection of Crimean-Congo hemorrhagic fever virus. Vector Borne Zoonotic Dis. 2012;12(9):786-93. doi: 10.1089/vbz.2011.0770.

(16) Midilli K, Gargili A, Ergonul O, Elevli M, Ergin S, Turan N, Sengöz G, Ozturk R, Bakar M. The first clinical case due to AP92 like strain of Crimean-Congo Hemorrhagic Fever virus and a field survey. BMC Infect Dis. 2009;9:90. doi: 10.1186/1471-2334-9-90.

(17) Midilli K, Gargili A, Ergonul O, Sengöz G, Ozturk R, Bakar M, Jongejan F. Imported Crimean-Congo hemorrhagic fever cases in Istanbul. BMC Infect Dis. 2007;7:54. doi: 10.1186/1471-2334-7-54.

Supplementary table 4. Number of ticks captured in each transect in the Natural Protected Areas of Doñana and Los Alcornocales from April 2018 to March 2019.

Transect[1]	*D. marginatus*		*H. punctata*				*Hy. lusitanicum*				*Hy. marginatum*		*I. acuminatus*	*I. ricinus*			*Rh. annulatus*		*Rh. bursa*			*Rh. pusillus*		*Rh. sanguineus*	
	M	F	L	N	M	F	L	N	M	F	L	F	M	N	M	F	L	N	L	M	F	M	F	M	F
D1	0	0	0	0	0	0	0	0	0	1	0	0	0	0	0	0	22	0	0	0	0	0	0	1	1
D2	0	0	0	0	0	0	0	2	0	1	0	0	0	0	0	0	1	0	0	0	0	0	0	0	0
D3	6	7	0	0	0	0	0	0	0	1	0	0	0	0	0	0	0	0	0	0	0	0	0	1	4
D4	0	1	0	0	0	0	0	44	5	8	0	0	0	0	0	0	0	0	0	0	0	0	0	1	1
D5	0	3	0	6	0	1	21	11	4	0	33	0	0	0	0	0	0	0	24	5	2	1	0	3	1
D6	0	0	0	1	0	0	0	3	0	0	0	0	0	0	0	0	74	0	0	11	4	0	0	2	0
D7	8	10	0	0	0	0	0	0	5	4	0	0	0	0	0	0	0	0	0	0	1	0	1	0	0
D8	0	0	0	0	0	0	142	39	5	4	0	0	0	0	1	0	29	1	0	0	0	0	0	0	0
D9	0	0	0	0	0	0	0	0	0	0	0	1	0	0	0	0	0	0	0	0	0	0	0	1	2
D10	1	0	0	0	0	0	0	0	0	0	0	0	0	0	0	0	0	0	0	0	0	0	0	1	0
D11	0	0	0	0	0	0	0	0	1	1	0	0	0	0	0	0	28	0	0	0	0	0	0	1	0
D12	0	0	0	0	0	0	0	0	3	3	0	0	0	0	0	0	0	0	0	0	0	0	0	0	0
D13	0	0	0	1	0	0	159	1	0	0	0	0	0	0	0	0	28	0	0	1	0	0	0	0	2
D14	0	0	0	0	0	0	0	0	1	0	0	0	0	0	0	0	0	0	0	1	0	0	0	0	0
D15	2	1	0	0	0	0	0	1	2	2	0	0	0	0	0	0	0	0	0	1	0	0	0	0	0
FD1	0	0	0	0	0	0	0	0	0	0	0	0	0	0	0	0	1	0	0	0	0	0	0	0	0
FD2	0	0	0	0	0	0	0	5	0	0	0	0	0	0	0	0	0	0	0	0	0	0	0	0	0
FD3	1	0	0	0	0	0	0	0	0	0	0	0	0	0	0	0	0	0	0	0	0	0	0	1	0
FD4	0	0	0	0	0	0	0	0	3	4	0	0	0	0	0	0	0	0	0	0	0	0	0	0	0
FD5	3	4	0	0	0	0	0	0	0	0	0	0	0	0	0	0	0	0	46	0	0	0	0	0	0
FD6	0	0	0	1	0	1	18	5	0	0	2	0	0	0	0	0	1	0	0	2	1	0	0	0	1
FD7	9	11	0	0	0	0	0	1	1	0	0	0	0	0	0	0	0	0	0	0	0	0	0	0	0
FD8	0	0	0	0	0	0	31	68	2	2	0	0	0	0	0	0	0	0	0	0	0	0	0	0	0
FD9	0	0	0	0	0	0	0	1	0	1	0	0	0	0	0	0	0	0	0	0	0	0	0	0	0
FD10	0	0	0	0	0	0	0	0	0	0	0	0	0	0	1	0	0	0	0	0	0	0	0	0	0
FD12	0	0	0	0	0	0	0	0	1	0	0	0	0	0	0	0	0	0	0	0	0	0	0	0	0
FD13	0	0	0	0	0	0	0	1	0	0	0	0	0	0	0	0	0	0	0	0	0	0	0	0	0
A1	0	1	0	0	0	0	0	17	30	19	0	0	0	0	0	0	0	0	0	0	0	2	6	0	0

Transect[1]	*D. marginatus*		*H. punctata*				*Hy. lusitanicum*				*Hy. marginatum*		*I. acuminatus*	*I. ricinus*			*Rh. annulatus*		*Rh. bursa*			*Rh. pusillus*		*Rh. sanguineus*	
	M	F	L	N	M	F	L	N	M	F	L	F	M	N	M	F	L	N	L	M	F	M	F	M	F
A2	**1**	0	0	**2**	0	0	0	**10**	**13**	**11**	0	0	0	0	**1**	**1**	0	0	**26**	**3**	**1**	0	0	0	0
A3	0	0	0	0	0	0	0	0	0	0	0	0	0	0	0	0	0	0	0	0	0	0	0	0	0
A4	0	0	0	0	0	0	0	0	0	0	0	0	0	0	0	0	0	0	0	0	0	0	0	**1**	0
A5	0	**1**	0	0	0	0	**2**	0	0	0	0	0	**1**	0	0	0	0	0	**21**	0	0	0	0	**4**	**3**
A6	0	**1**	0	0	0	0	**31**	**9**	**10**	**3**	0	0	0	0	0	**1**	0	0	**1**	**2**	**1**	0	0	**3**	**4**
A7	0	0	**143**	0	**1**	**1**	**22**	**1**	0	0	0	0	0	0	**1**	0	0	0	0	0	0	0	0	**1**	0
A8	0	0	**176**	**3**	**1**	0	**2**	**5**	**2**	**1**	0	0	0	**1**	0	0	0	0	**2**	**5**	**3**	0	0	**1**	**1**
A9	0	0	0	0	**1**	0	**213**	**4**	0	**1**	0	0	0	0	0	0	**4**	0	**28**	**1**	**1**	0	**1**	0	0
A10	0	0	0	0	0	0	0	0	0	0	0	0	0	0	0	0	0	0	0	0	0	0	0	0	0
A11	0	0	0	0	0	0	**63**	**2**	**1**	0	0	0	0	0	0	0	**3**	0	**4**	0	0	0	0	**3**	**4**
A12	0	0	0	0	0	0	0	0	0	0	0	0	0	0	0	0	0	0	0	0	0	0	0	0	0
A13	0	0	0	0	0	0	**236**	**6**	0	**1**	0	0	0	0	0	0	0	0	0	**5**	**11**	0	0	0	**1**
A14	0	0	0	0	0	0	**152**	**20**	**6**	**5**	0	0	0	0	0	0	0	0	**32**	**1**	**4**	0	0	**1**	**3**
A15	0	0	0	**1**	0	0	0	**1**	0	0	0	0	0	0	0	0	0	0	0	0	0	0	0	0	0
A16	0	0	0	**49**	**26**	**17**	**1223**	**37**	**280**	**258**	0	0	0	0	**1**	0	0	0	0	0	0	0	0	0	0
FA5	0	0	0	0	0	0	0	0	0	0	0	0	0	0	0	0	0	0	**6**	**1**	0	0	0	0	**2**
FA6	0	0	0	0	**1**	0	0	0	**1**	**5**	0	0	0	0	0	0	0	0	**2**	0	0	0	0	0	0
FA7	0	0	0	0	0	0	0	0	0	0	0	0	0	0	0	0	0	0	0	0	0	0	0	0	0
FA8	0	0	0	0	0	0	**33**	0	0	0	0	0	0	0	0	0	0	0	0	**2**	**1**	0	0	0	0
FA9	0	0	0	0	**1**	0	0	**53**	0	0	0	0	0	0	0	0	0	0	**37**	0	0	0	0	0	0
FA11	0	0	0	0	0	0	0	**2**	0	0	0	0	0	0	0	0	3	0	**8**	0	0	0	0	0	0
FA13	0	0	0	0	0	0	0	0	0	0	0	0	0	0	0	0	0	0	**60**	0	0	0	0	0	0
FA14	0	0	0	0	0	0	**15**	**14**	0	0	0	0	0	0	0	0	**91**	0	**0**	**9**	**8**	0	0	0	0

[1]D1-D15: Transects in the Natural Protected Area of Doñana; FD: Buffer areas in the NPAD; A1-A16: Transects in the Natural Protected Area of Los Alcornocales; FA: Buffer areas in the NPAA; *D.*: *Dermacentor*; *H.*: *Haemaphysalis*; *Hy*.: *Hyalomma*; *I.: Ixodes*; *Rh.: Rhipicephalus*; L: Larvae; N: Nymph; M: male; F: Female

Supplementary table 5. Number of ticks captured in each transect in April 2018 in the Natural Protected Areas of Doñana and Los Alcornocales.

Transect1	*D. marginatus*		*H. punctata*				*Hy. lusitanicum*				*Hy. marginatum*		*I. acuminatus*	*I. ricinus*			*Rh. annulatus*		*Rh. bursa*			*Rh. pusillus*		*Rh. sanguineus*	
	M	F	L	N	M	F	L	N	M	F	L	F	M	N	M	F	L	N	L	M	F	M	F	M	F
D1										1														1	1
D2																									
D3	2	4																						1	2
D4									1	1															
D5		1							1															1	
D6																									
D7																									
D8																									
D9												1												1	
D10	1																							1	
D11																									
D12									2	2															
D13																									2
D14																									
D15																									
A1									14	7													5		
A2																									
A3																									
A4																									
A5		1																							
A6																								2	2
A7					1	1									1									1	
A8					1																				
A9																									
A10																									
A11																									
A12																									
A13																									
A14																									

[1]D1-D15: Transects in the Natural Protected Area of Doñana; A1-A16: Transects in the Natural Protected Area of Los Alcornocales ; *D.*: *Dermacentor*; *H.*: *Haemaphysalis*; *Hy.*: *Hyalomma*; *I.: Ixodes*; *Rh.: Rhipicephalus*; L: Larvae; N: Nymph; M: male; F: Female

Supplementary table 6. Number of ticks captured in each transect in May 2018 in the Natural Protected Area of Doñana and in June 2018 in the Natural Protected Area of Los Alcornocales.

Transect[1]	*D. marginatus*		*H. punctata*				*Hy. lusitanicum*				*Hy. marginatum*		*I. acuminatus*	*I. ricinus*			*Rh. annulatus*		*Rh. bursa*			*Rh. pusillus*		*Rh. sanguineus*	
	M	F	L	N	M	F	L	N	M	F	L	F	M	N	M	F	L	N	L	M	F	M	F	M	F
D1																									
D2										1															
D3	2	2								1															1
D4									4	3														1	1
D5									2											3	2	1		1	1
D6																					1			2	
D7									5	4											1				
D8									2	1							29								
D9																									2
D10																									
D11									1	1														1	
D12									1	1															
D13																				1					
D14									1											1					
D15										1										1					
A1									6	3												1			
A2									12	9															
A4																								1	
A5																								4	3
A6									8	3														1	1
A7			143																						
A8			169				1		2	1				1						3				1	1
A9										1											1		1		
A11									1															3	4
A12																									
A13																				1	2				1

[1]D1-D15: Transects in the Natural Protected Area of Doñana; A1-A16: Transects in the Natural Protected Area of Los Alcornocales; *D.*: *Dermacentor*; *H.*: *Haemaphysalis*; *Hy.*: *Hyalomma*; *I.*: *Ixodes*; *Rh.*: *Rhipicephalus*; L: Larvae; N: Nymph; M: male; F: Female

Supplementary table 7. Number of ticks captured in each transect in August 2018 in the Natural Protected Areas of Doñana and Los Alcornocales.

Transect[1]	*D. marginatus*		*H. punctata*				*Hy. lusitanicum*				*Hy. marginatum*		*I. acuminatus*	*I. ricinus*			*Rh. annulatus*		*Rh. bursa*			*Rh. pusillus*		*Rh. sanguineus*	
	**M**	**F**	**L**	**N**	**M**	**F**	**L**	**N**	**M**	**F**	**L**	**F**	**M**	**N**	**M**	**F**	**L**	**N**	**L**	**M**	**F**	**M**	**F**	**M**	**F**
D1																	22							1	1
D2								2									1								
D3																								1	4
D4								3		3														1	1
D5				6			21	11												2				3	1
D6				1				3												11	3			2	
D7																							1		
D8							142	39	1	3								1							
D9																								1	2
D10																								1	
D11																	28							1	
D12																									
D13				1			159	1									20								2
D15									2	1															
FD4																									
FD6				1			18	4												2	1				
FD8							31	67	2	2														1	
A1								17	7	7												1	1		
A2				1				7		1										3					
A5																									1
A6									1											2	1				
A7							22																		
A8			7	3				2											2	2	3				
A9							213													1					
A11							62	1																	
A13							236			1										4	9				
A14							152														2				
A16							1067	8	134	147															
FA5																				1					

Transect[1]	*D. marginatus*		*H. punctata*				*Hy. lusitanicum*				*Hy. marginatum*		*I. acuminatus*	*I. ricinus*			*Rh. annulatus*		*Rh. bursa*			*Rh. pusillus*		*Rh. sanguineus*	
	**M**	**F**	**L**	**N**	**M**	**F**	**L**	**N**	**M**	**F**	**L**	**F**	**M**	**N**	**M**	**F**	**L**	**N**	**L**	**M**	**F**	**M**	**F**	**M**	**F**
FA6									1	5														1	
FA8							33													2	1			4	3
FA9																								3	4
FA14							15													9	8			1	

[1]D1-D15: Transects in the Natural Protected Area of Doñana; FD: Buffer areas in Doñana; A1-A16: Transects in the Natural Protected Area of Los Alcornocales; FA: Buffer areas in Alcornocales

D.: *Dermacentor*; *H*.: *Haemaphysalis*; *Hy*.: *Hyalomma*; *I.*: *Ixodes*; *Rh.*: *Rhipicephalus*; L: Larvae; N: Nymph; M: male; F: Female

Supplementary table 8. Number of ticks captured in each transect in October 2018 in the Natural Protected Areas of Doñana and Los Alcornocales.

Transect[1]	*D. marginatus*		*H. punctata*				*Hy. lusitanicum*				*Hy. marginatum*		*I. acuminatus*	*I. ricinus*			*Rh. annulatus*		*Rh. bursa*			*Rh. pusillus*		*Rh. sanguineus*	
	M	F	L	N	M	F	L	N	M	F	L	F	M	N	M	F	L	N	L	M	F	M	F	M	F
D1																								1	1
D2																								0	0
D3																								1	4
D4								41		1														1	1
D5											33													3	1
D6																	74							2	0
D7																								0	0
D8									1															0	0
D9																								1	2
D10																								1	0
D11																								1	0
D12																								0	0
D13																								0	2
D15								1																0	0
FD1																	1							0	0
FD2								5																0	0
FD4									3	2														0	0
FD6								1			2						1							1	0
FD7								1																0	0
FD8								1																0	0
FD9								1		1														0	1
FD12									1															0	0
FD13								1																0	0
A1									3	1														0	0
A2				1				3		1											1			0	0
A5							2																	0	0
A6							31	9																0	0
A7								1																0	0
A8							1	3																0	0

Transect[1]	***D. marginatus***		***H. punctata***				***Hy. lusitanicum***				***Hy. marginatum***		***I. acuminatus***	***I. ricinus***			***Rh. annulatus***		***Rh. bursa***			***Rh. pusillus***		***Rh. sanguineus***	
	**M**	**F**	**L**	**N**	**M**	**F**	**L**	**N**	**M**	**F**	**L**	**F**	**M**	**N**	**M**	**F**	**L**	**N**	**L**	**M**	**F**	**M**	**F**	**M**	**F**
A9								4									4							0	0
A11							1	1									3							1	0
A13								6																4	3
A14								20																3	4
A15				1				1																1	0
A16				10			156	29	104	83														1	1
FA9								51																0	0
FA11								2									3							0	0
FA14								14									91							3	4

[1]D1-D15: Transects in the Natural Protected Area of Doñana; FD: Buffer areas in Doñana; A1-A16: Transects in the Natural Protected Area of Los Alcornocales; FA: Buffer areas in Alcornocales; *D.*: *Dermacentor*; *H.*: *Haemaphysalis*; *Hy.*: *Hyalomma*; *I.: Ixodes*; *Rh.: Rhipicephalus*; L: Larvae; N: Nymph; M: male; F: Female

Supplementary table 9. Number of ticks captured in each transect in November 2018 in the Natural Protected Area of Doñana and in December 2018 in the Natural Protected Area of Los Alcornocales.

Transect[1]	*D. marginatus*		*H. punctata*				*Hy. lusitanicum*				*Hy. marginatum*		*I. acuminatus*	*I. ricinus*			*Rh. annulatus*		*Rh. bursa*			*Rh. pusillus*		*Rh. sanguineus*	
	M	F	L	N	M	F	L	N	M	F	L	F	M	N	M	F	L	N	L	M	F	M	F	M	F
D1																									
D2																									
D3																									
D4		1																							
D5		2				1													24						
D6																									
D7	8	9																							
D8																									
D9																									
D10																									
D11																									
D12																									
D13																									
D15	2	1																							
FD4										1															
FD5		1																	46						
FD6						1																			
FD7	4	6																							
A1																									
A2																1			26						
A5																			7						
A6																									
A7																									
A8																									
A9					1														28						
A11																			4						
A13																									
A14																			5						

Transect[1]	*D. marginatus*		*H. punctata*				*Hy. lusitanicum*				*Hy. marginatum*		*I. acuminatus*	*I. ricinus*			*Rh. annulatus*		*Rh. bursa*			*Rh. pusillus*		*Rh. sanguineus*	
	**M**	**F**	**L**	**N**	**M**	**F**	**L**	**N**	**M**	**F**	**L**	**F**	**M**	**N**	**M**	**F**	**L**	**N**	**L**	**M**	**F**	**M**	**F**	**M**	**F**
A15																									
A16				6	8	4			21	14					1										
FA9					1			2											37						
FA11																			8						
FA13																			57						

[1]D1-D15: Transects in the Natural Protected Area of Doñana; FD: Buffer areas in Doñana; A1-A16: Transects in the Natural Protected Area of Los Alcornocales; FA: Buffer areas in Alcornocales; *D*.: *Dermacentor*; *H*.: *Haemaphysalis*; *Hy*.: *Hyalomma*; *I.: Ixodes*; *Rh.: Rhipicephalus*; L: Larvae; N: Nymph; M: male; F: Female

Supplementary table 10. Number of ticks captured in each transect in February 2019 in the Natural Protected Areas of Doñana and Los Alcornocales.

Transect[1]	*D. marginatus*		*H. punctata*				*Hy. lusitanicum*				*Hy. marginatum*		*I. acuminatus*	*I. ricinus*			*Rh. annulatus*		*Rh. bursa*			*Rh. pusillus*		*Rh. sanguineus*	
	**M**	**F**	**L**	**N**	**M**	**F**	**L**	**N**	**M**	**F**	**L**	**F**	**M**	**N**	**M**	**F**	**L**	**N**	**L**	**M**	**F**	**M**	**F**	**M**	**F**
D1																									
D2																									
D3	1	1																							
D4																									
D5																									
D6																									
D7		1																							
D8															1										
D9																									
D10																									
D11																									
D12																									
D13																	8								
D15																									
FD5	3	2																							
FD7	5	5																							
FD10															1										
A1		1																							
A2															1										
A5													1						14						
A6																			1						
A7																									
A8																									
A9																									
A11																									
A13																									
A14																			27						
A15																									
A16				33	18	13			21	14															

Transect[1]	*D. marginatus*		*H. punctata*				*Hy. lusitanicum*				*Hy. marginatum*		*I. acuminatus*	*I. ricinus*			*Rh. annulatus*		*Rh. bursa*			*Rh. pusillus*		*Rh. sanguineus*	
	**M**	**F**	**L**	**N**	**M**	**F**	**L**	**N**	**M**	**F**	**L**	**F**	**M**	**N**	**M**	**F**	**L**	**N**	**L**	**M**	**F**	**M**	**F**	**M**	**F**
FA5																			6						
FA6																			2						
FA7					1																				
FA13																			3						

[1]D1-D15: Transects in the Natural Protected Area of Doñana; FD: Buffer areas in Doñana; A1-A16: Transects in the Natural Protected Area of Los Alcornocales; FA: Buffer areas in Alcornocales;

D.: *Dermacentor*; *H*.: *Haemaphysalis*; *Hy*.: *Hyalomma*; *I.: Ixodes*; *Rh.: Rhipicephalus*; L: Larvae; N: Nymph; M: male; F: Female

Supplementary table 11. Number of ticks captured in each transect in March 2019 in the Natural Protected Areas of Doñana and Los Alcornocales.

Transect[1]	*D. marginatus*		*H. punctata*				*Hy. lusitanicum*				*Hy. marginatum*		*I. acuminatus*	*I. ricinus*			*Rh. annulatus*		*Rh. bursa*			*Rh. pusillus*		*Rh. sanguineus*	
	M	F	L	N	M	F	L	N	M	F	L	F	M	N	M	F	L	N	L	M	F	M	F	M	F
D1																									
D2																									
D3	1																								1
D4																									
D5									1															1	
D6																									
D7																									
D8									1																
D9																									
D10																									
D11																									
D12																									
D13																									
D15																									
FD3	1																							1	
FD4										1															
FD5		1																							
FD6																									1
FD7									1																
A1										1															
A2	1								1																
A5																									
A6		1							1							1									1
A7																									
A8																									
A9																									
A11																									
A13																									
A14																									

Transect[1]	*D. marginatus*		*H. punctata*				*Hy. lusitanicum*				*Hy. marginatum*		*I. acuminatus*	*I. ricinus*			*Rh. annulatus*		*Rh. bursa*			*Rh. pusillus*		*Rh. sanguineus*	
	**M**	**F**	**L**	**N**	**M**	**F**	**L**	**N**	**M**	**F**	**L**	**F**	**M**	**N**	**M**	**F**	**L**	**N**	**L**	**M**	**F**	**M**	**F**	**M**	**F**
A15																									
A16																									

[1]D1-D15: Transects in the Natural Protected Area of Doñana; FD: Buffer areas in Doñana; A1-A16: Transects in the Natural Protected Area of Los Alcornocales; FA: Buffer areas in Alcornocales;

D.: *Dermacentor*; *H*.: *Haemaphysalis*; *Hy*.: *Hyalomma*; *I.: Ixodes*; *Rh.: Rhipicephalus*; L: Larvae; N: Nymph; M: male; F: Female

Supplementary table 12. Tick densities in each transect from April 2018 to March 2019 in the Natural Protected Areas of Doñana and Los Alcornocales.

Transect[1]	Apr-18	May-18 Jun-18	Aug-18	Oct-18	Nov-18 Dec-18	Feb-19	Mar-19	Total No. of ticks	No. of samplings	No. of ticks per 100 m^2
D1	3	0	22	0	0	0	0	25	7	3.6
D2	0	1	3	0	0	0	0	4	7	0.6
D3	9	6	0	0	0	2	2	19	7	2.7
D4	2	9	6	42	1	0	0	60	7	8.6
D5	3	10	40	33	27	0	2	115	7	16.4
D6	0	3	18	74	0	0	0	95	7	13.6
D7	0	10	1	0	17	1	0	29	7	4.1
D8	0	32	186	1	0	1	1	221	7	31.6
D9	2	2	0	0	0	0	0	4	7	0.6
D10	2	0	0	0	0	0	0	2	7	0.3
D11	0	3	28	0	0	0	0	31	7	4.4
D12	4	2	0	0	0	0	0	6	7	0.9
D13	2	1	181	0	0	8	0	192	7	27.4
D14	0	2						2	2	1.0
D15	0	2	3	1	3	0	0	9	7	1.3
FD1				1				1	1	1.0
FD2				5				5	1	5.0
FD3							2	2	1	2.0
FD4			0	5	1		1	7	4	1.8
FD5					47	5	1	53	3	17.7
FD6			26	4	1		1	32	4	8.0
FD7				1	10	10	1	22	4	5.5
FD8			102	1				103	2	51.5
FD9				2				2	1	2.0
FD10						1		1	1	1.0
FD12				1				1	1	1.0

Transect[1]	Apr-18	May-18 Jun-18	Aug-18	Oct-18	Nov-18 Dec-18	Feb-19	Mar-19	Total No. of ticks	No. of samplings	No. of ticks per 100 m^2
FD13				1				1	1	1.0
A1	26	10	33	4		1	1	75	7	10.7
A2	0	21	12	6	27	1	2	69	7	9.9
A3	0							0	1	0.0
A4	0	1						1	2	0.5
A5	1	7	0	2	7	15	0	32	7	4.6
A6	4	13	4	40	0	1	4	66	7	9.4
A7	4	143	22	1	0	0	0	170	7	24.3
A8	1	179	19	4	0	0	0	203	7	29.0
A9	0	3	214	8	29	0	0	254	7	36.3
A10	0							0	1	0.0
A11	0	8	63	5	4	0	0	80	7	11.4
A12	0	0						0	2	0.0
A13	0	4	250	6	0	0	0	260	7	37.1
A14	0	18	154	20	5	27	0	224	7	32.0
A15				2	0	0	0	2	4	0.5
A16			1356	382	54	99	0	1891	5	378.2
FA5			3			6	0	9	3	3.0
FA6			6			2	0	8	3	2.7
FA7						1	0	1	2	0.5
FA8			36				0	36	2	18.0
FA9			0	51	40		0	91	4	22.8
FA11				5	8		0	13	3	4.3
FA13					57	3	0	60	2	30.0
FA14			32	105			0	137	3	45.7

[1]AD1-D15: Transects in the Natural Protected Area of Doñana; FD: Buffer areas in Doñana; A1-A16: Transects in the Natural Protected Area of Los Alcornocales; FA: Buffer areas in Alcornocales

Supplementary table 13. Temperature and relative humidity during tick surveys in Doñana.

Sampling	Temperature (ºC)			Relative humidity (%)			Daytime
	Minimum	Maximum	Mean (CI)	Minimum	Maximum	Mean (CI)	
S-1; Apr-18	16	22	19.5 (16.7-22.2)	41	65	51.5 (48.7-54.2)	10.45 am-4.00 pm
S-2; May-18	20.2	27.5	22.8 (20-25.5)	44	65	54.3 (51.6-57.1)	10.45 am-4.15 pm
S-3; Aug-18	22.5	39.9	32.1 (29.6-34.7)	21	73	37.6 (35.1-40.2)	8.55 am-1.50 pm
S-4; Oct-18	22.7	33.3	28.4 (25.7-31.2)	22	82	39.4 (36.6-42.2)	10.00 am-3.05 pm
S-5; Nov-18	10.6	19.7	14.8 (12.3-17.2)	58	90	68.9 (66.4-71.4)	10.50 am-2.15 pm
S-6; Feb-19	11.2	23.0	15.5 (12.9-18.1)	34	71	50.9 (48.4-53.5)	10.50 am-5.35 pm
S-7; Mar-19	14.9	26.0	20.4 (17.9-22.9)	29	66	48.1 (45.6-50.5)	10.30 am-5.05 pm

Supplementary table 14. Temperature and relative humidity during tick surveys in Alcornocales.

Sampling	Temperature (ºC)			Relative humidity (%)			Daytime
	Minimum	Maximum	Mean (CI)	Minimum	Maximum	Mean (CI)	
S-1; Apr-18	17.2	25.5	20.9 (18.0-23.8)	43	67	52.1 (49.2-55.0)	11.15 am-4.05 pm
S-2; May-18	20.5	26.2	22.6 (19.4-25.8)	47	69	55.6 (52.4-58.8)	11.15 am-3.40 pm
S-3; Aug-18	28.0	37.8	32.1 (29.2-35.0)	27	53	37.6(34.8-40.5)	9.45 am-2.20 pm
S-4; Oct-18	20.5	29.9	24.3 (21.5-27.1)	32	82	61.7 (59.0-64.5)	10.02 am-3.17 pm
S-5; Nov-18	16.1	25.7	21.2 (18.5-23.8)	44	86	62.4 (59.8-65.1)	10.30 am-3.37 pm
S-6; Feb-19	14.6	17.5	15.9 (13.2-18.6)	34	85	73.3 (70.8-76.1)	10.47 am-3.47 pm
S-7; Mar-19	15.8	21.1	18.5 (15.3-21.7)	38	81	53.5 (50.3-56.6)	10.25 am-2.45 pm

Supplementary table 15. Maximum Identities of the sequences obtained in this study comparing with public sequences of GenBank.

Organisms	Target genes	Species	No. of sequences	GenBank acc. No.	Identities			
					bp	%	GenBank Acc. No.	QC
**Ticks**	16S rRNA	*Haemaphysalis punctata*	1	PZ060376	387/388	99.7	MF002566*	100
			3	PZ060377	404/404	100	MG021187*	100
		Hyalomma lusitanicum	26	PZ060378	402/402	100	ON366982	100
			1		402/403	99.8	ON366982*	100
				PZ060379	392/392	100	LC508317	97
			1	PZ060380	401/403	99.5	ON366982*	100
			1	PZ060381	401/403	99.5	ON366982*	100
		Hyalomma marginatum	1	PZ060382	403/404	99.8	MW366630*	100
			2	PZ060383	403/403	100	MW366630*	100
		Ixodes acuminatus	1	PZ060384	407/409	99.5	PQ144653	100
		Ixodes ricinus	1	PZ060385	406/407	100	GU074598*	100
			1	PZ060386	390/390	100	OP375394*	100
**-**		*Rhipicephalus annulatus*	4	PZ060387	402/403	99.8	L34311*	100
		Rhipicephalus bursa	2	PZ060388	401/401	100	OR773535*	100
		Rhipicephalus pusillus	1	PZ060389	405/408	99.3	OL454855*	100
		Rhipicephalus sanguineus sensu stricto	2	PZ060390	384/384	100	OR946410	100
			2	PZ060391	412/412	100	JX997393*	100
Borrelia	*flaB*	*Borrelia lusitaniae*	6	PZ112783	453/453<br>391/391	100	CP132468	100
Rickettsia	*ompA*	*Candidatus* Rickettsia rioja	39	PZ112789	488/488	100	GQ404429*	100
			2	PZ112790	486/488	99.6	GQ404429*	100
			2	PZ112791	487/488	99.8	GQ404429*	100
		Rickettsia aeschlimannii	2	PZ112792	588/588	100	HQ335157*	100
		Rickettsia barbariae	4	PZ112793	583/583<br>488/488	100	MK028340*	100
		Rickettsia massiliae	16	PZ112794	590/590<br>490/490	100	CP000683*	100
			2	PZ112795	448/448	100	CP003319*	100
		Rickettsia monacensis	5	PZ112796	598/603<br>590/590	100	LN794217*<br>PP176516	100<br>98
		Rickettsia raoultii	1	PZ112797	587/587	100	AH015609	100

Organisms	Target genes	Species	No. of sequences	GenBank acc. No.	Identities			
					**bp**	**%**	**GenBank Acc. No.**	**QC**
		Rickettsia slovaca	14	PZ112798	588/588<br>491/491	100	CP002428	100
	ompB	*R. barbariae*	3	PZ112799	382/382	100	MK028339*	100
		R. massiliae	2	PZ112800	483/483	100	CP000683*	100
			3	PZ112801	483/483	100	CP003319*	100
	Sca4	*R. barbariae*	2	PZ112802	803/803	100	PP954858*	100
Ehrlichia	gro*ESL*		5	PZ112784	1308/1310	99.9	KJ930191	98
					1089/1089	100	OQ185227	82
			2	PZ112785	705/705	100	KJ930193*	100
	16S rRNA		4	PZ059407	659/659	100	AF414399*	100
	gltA		4	PZ112786	920/920	100	KX987353*	100
			2	PZ112787	848/920	92.2	AF311965	100
			1	PZ112788	646/647	99.9	OQ185264*	100

* among others
