# Supplementary material for: Surveillance of ticks and tick-borne microorganisms in two natural protected areas in Andalusia, South of Spain
Source: Parasit Vectors. 2026 Aug 10;19:476. doi: 10.1186/s13071-026-07580-1 (PMC13737913; doi:10.1186/s13071-026-07580-1)
Supplement: Supplementary file 2 [file 13071_2026_7580_MOESM2_ESM.pdf]

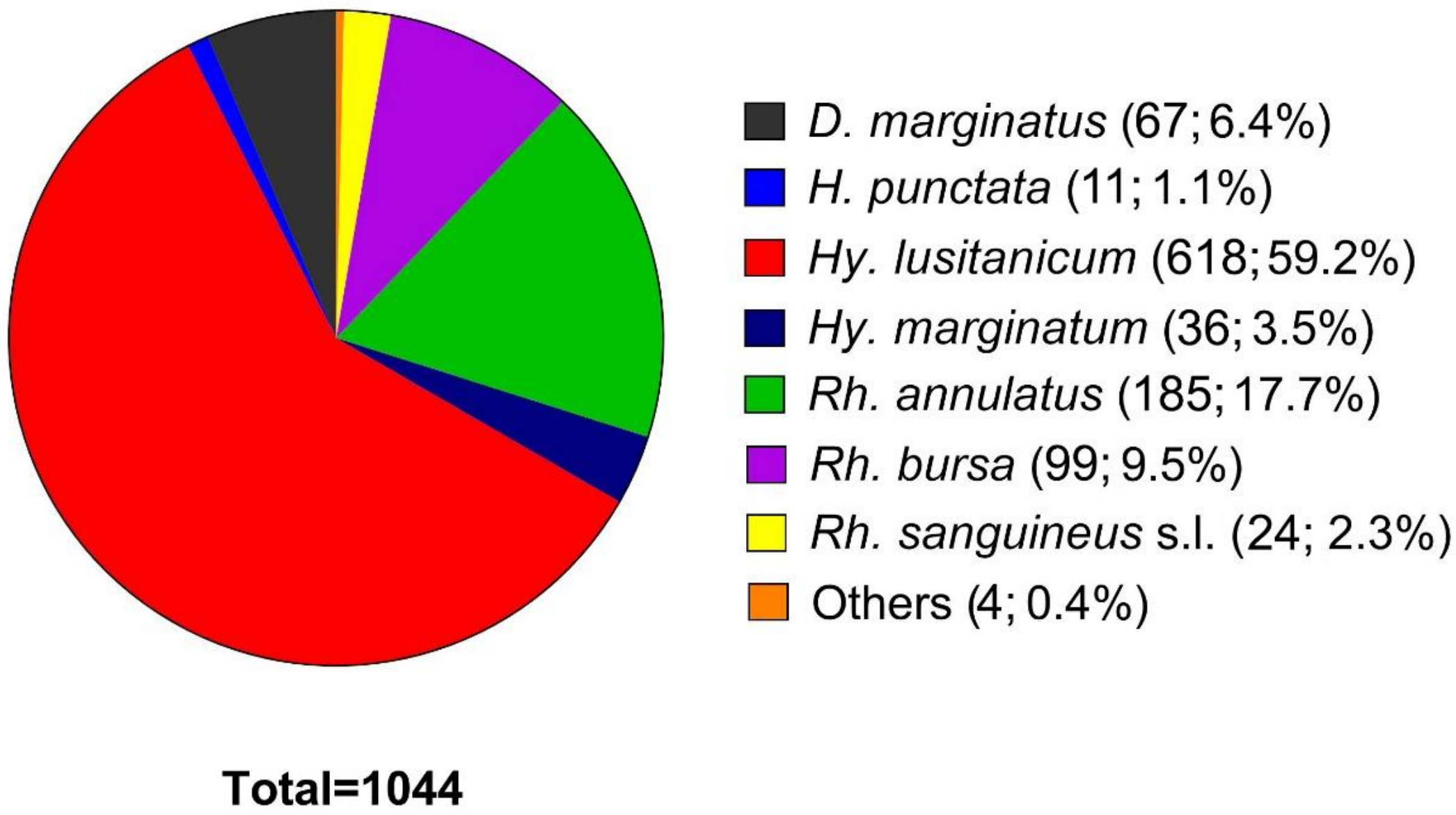

Supplementary figure 1: Abundance of tick species captured in the Natural Protected Area of Doñana from April 2018 to March 2019. 'Others' correspond to species with a prevalence <1%, specifically to *Ixodes ricinus* (0.2%) and *Rhipicephalus pusillus* (0.2%). *D.: Dermacentor; H.: Haemaphysalis; Hy: Hyalomma; Rh.: Rhipicephalus*.

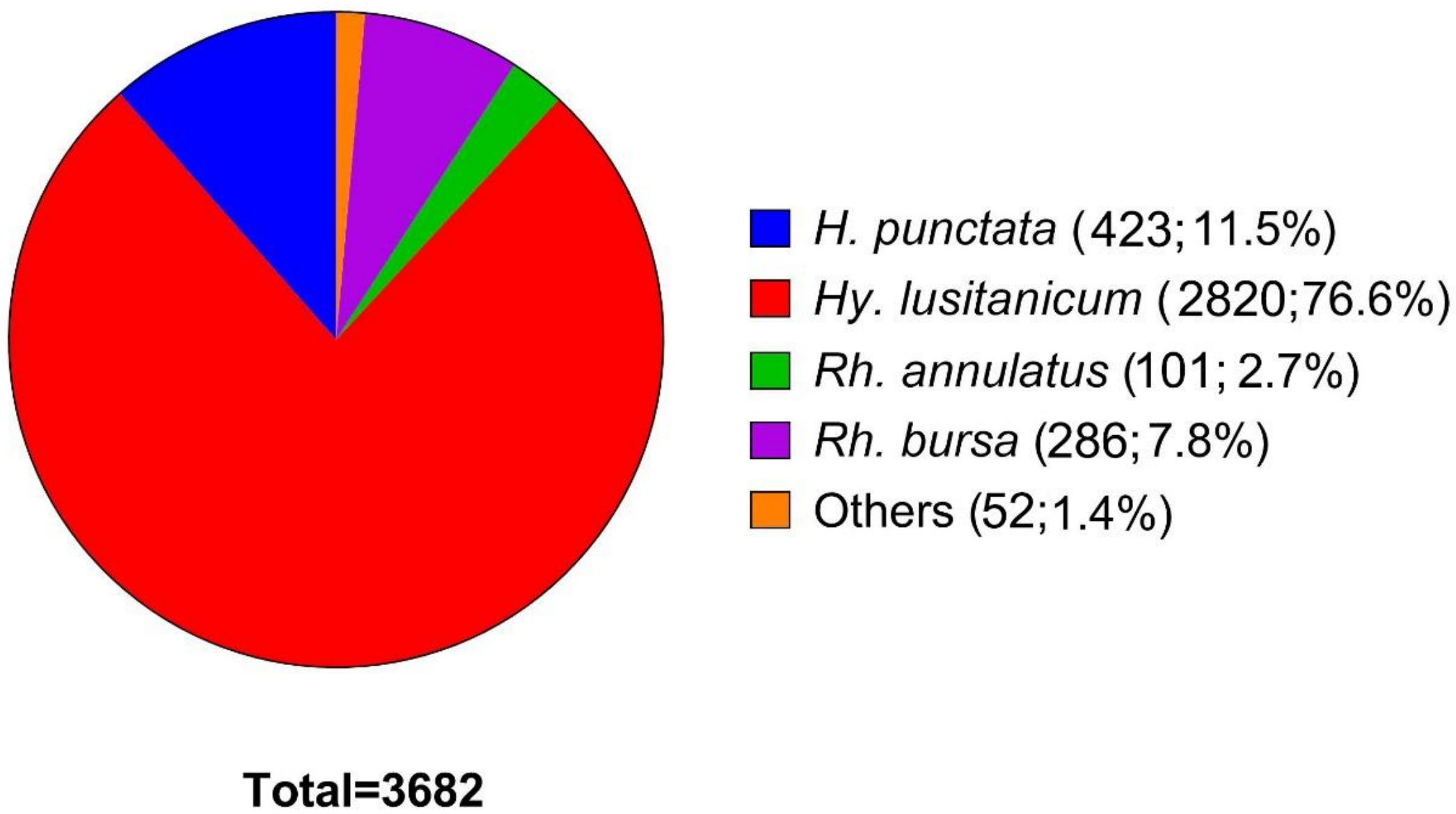

Supplementary figure 2: Abundance of tick species captured in the Natural Protected Area of Los Alcornocales from April 2018 to March 2019. 'Others' correspond to species with a prevalence <1%, specifically to *Dermacentor marginatus* (0.1%), *Ixodes acuminatus* (0.03%), *Ixodes ricinus* (0.2%), *Rhipicephalus pusillus* (0.2%) and *Rhipicephalus sanguineus* sensu lato (0.9%). *H.: Haemaphysalis; Hy: Hyalomma; Rh.: Rhipicephalus*.

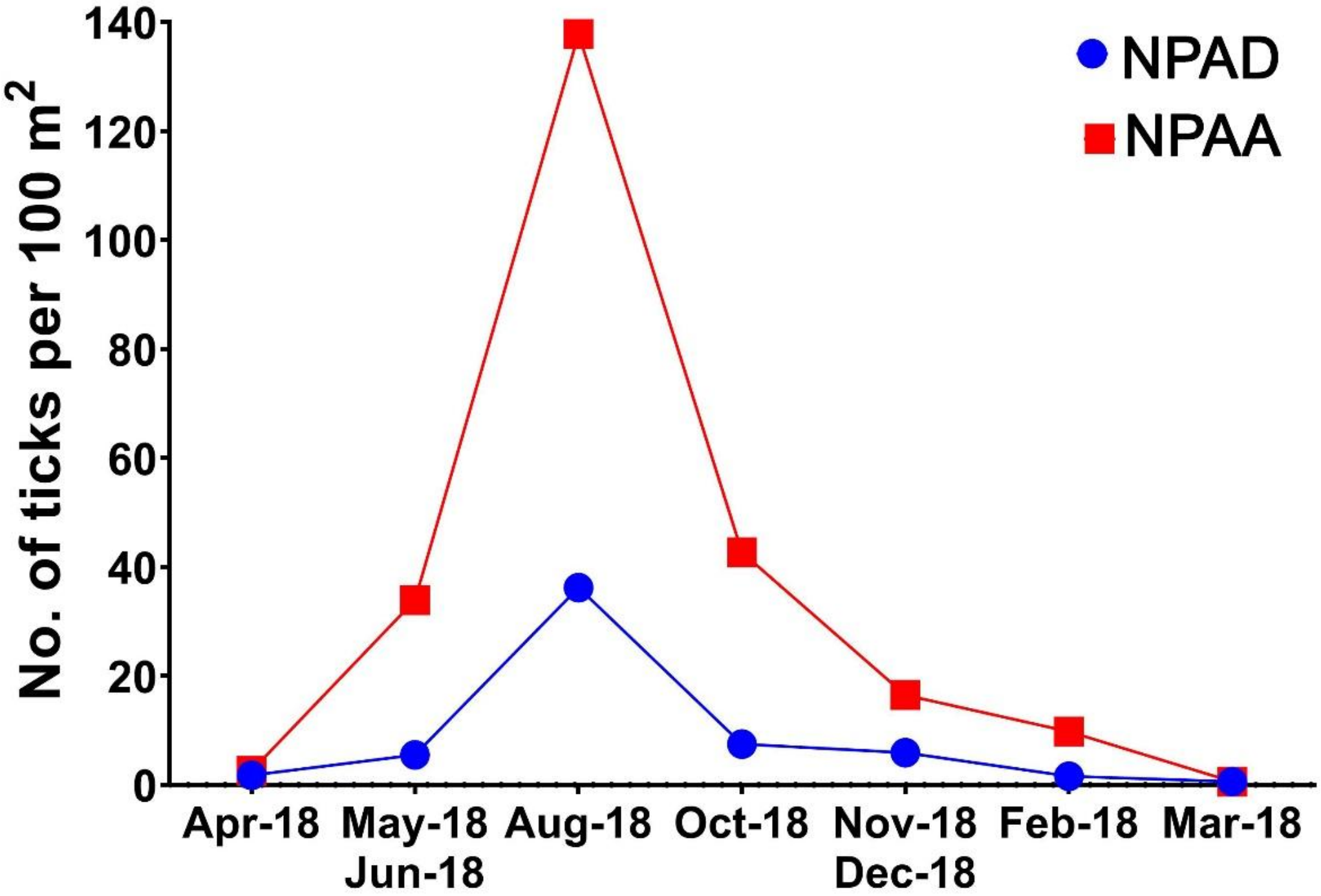

Supplementary figure 3: Abundance of tick specimens in the seven samplings performed in the Natural Protected Area of Doñana (NPAD) and in the Natural Protected Area of Los Alcornocales (NPAA).

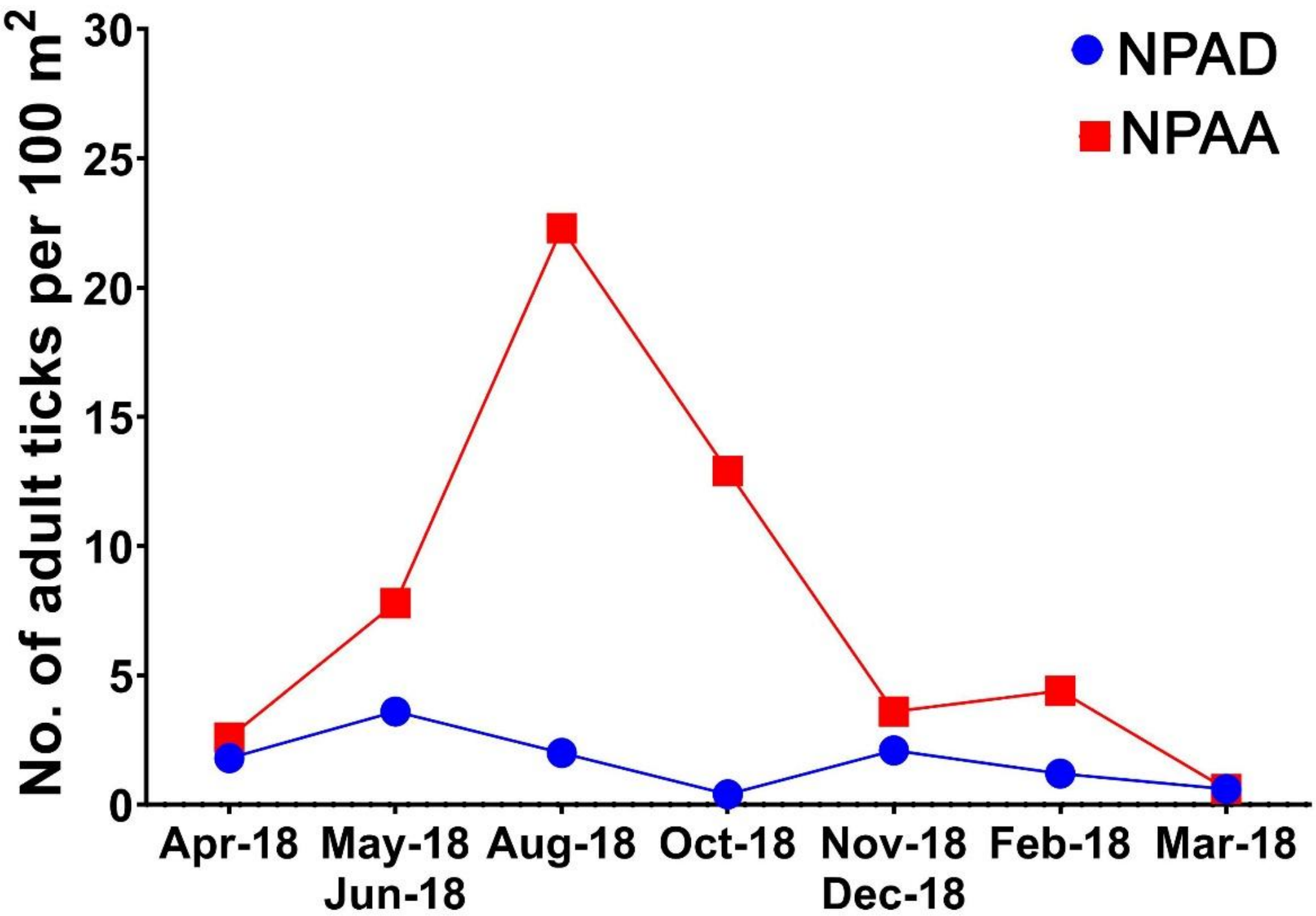

Supplementary figure 4: Abundance of adult tick specimens in the seven samplings performed in the Natural Protected Area of Doñana (NPAD) and in the Natural Protected Area of Los Alcornocales (NPAA)

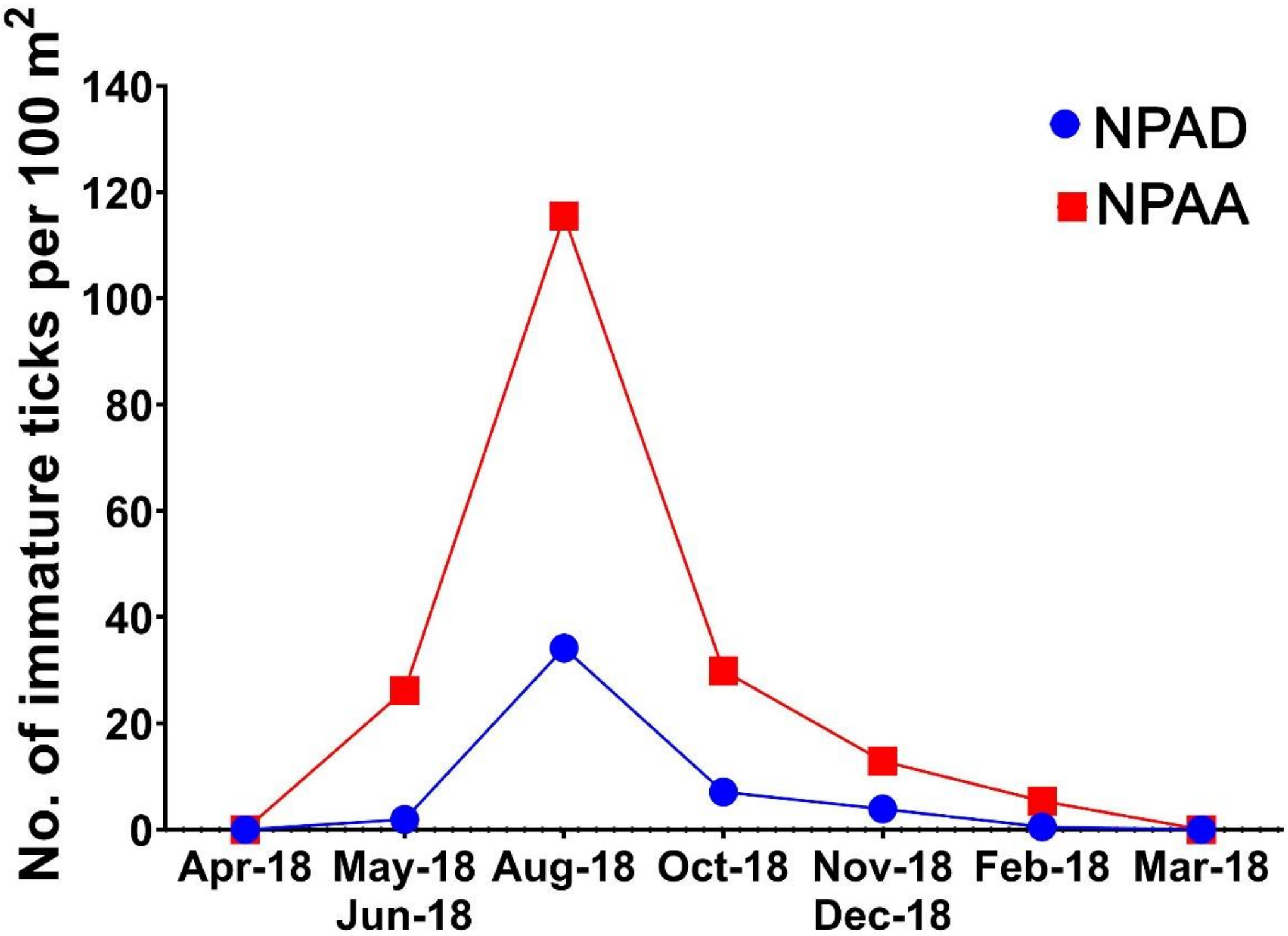

Supplementary figure 5: Abundance of immature tick specimens in the seven samplings performed in the Natural Protected Area of Doñana (NPAD) and in the Natural Protected Area of Los Alcornocales (NPAA).

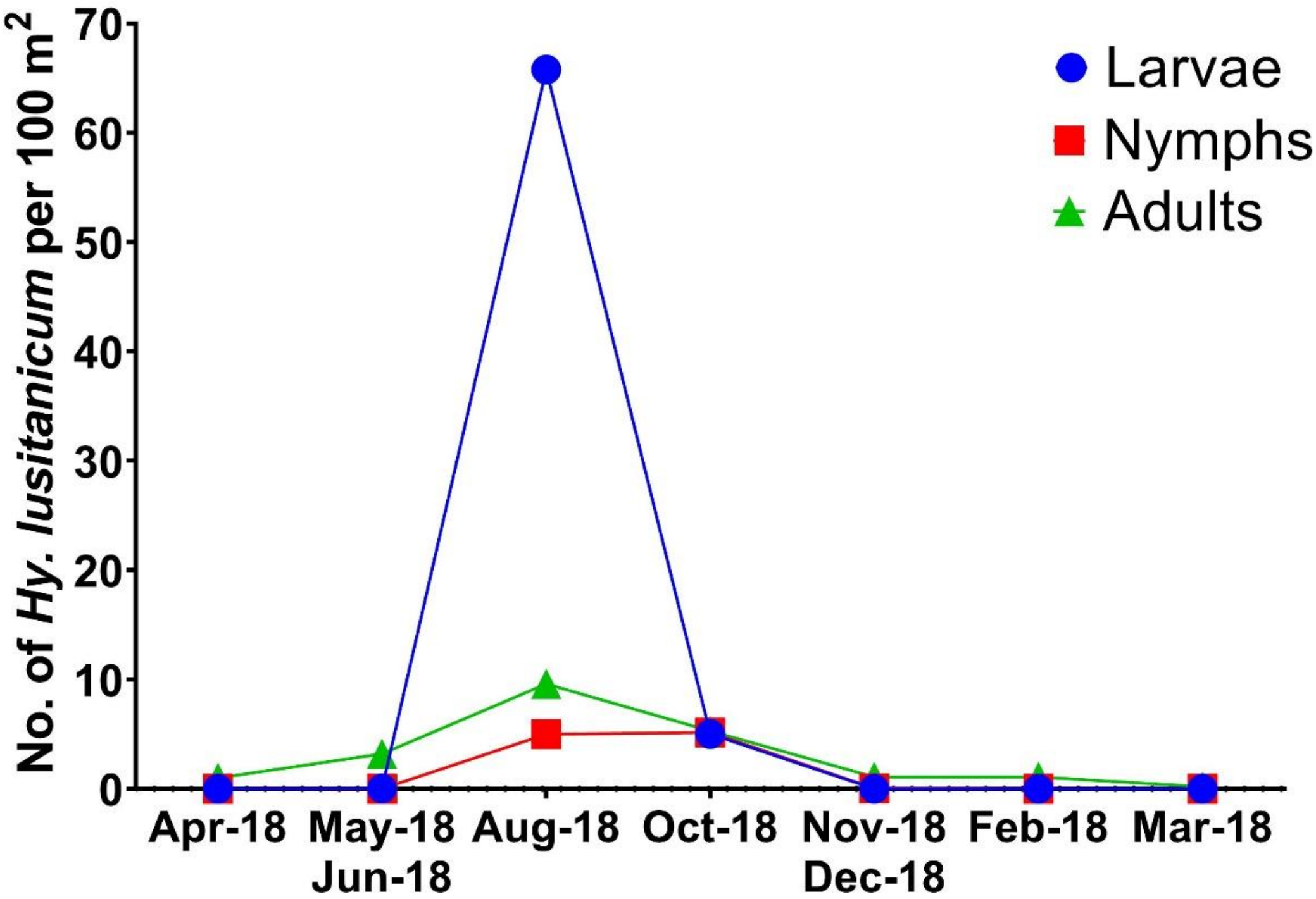

Supplementary figure 6: Abundance of *Hyalomma lusitanicum* tick specimens in the seven samplings performed in the Natural Protected Area of Doñana (NPAD) and in the Natural Protected Area of Los Alcornocales (NPAA).

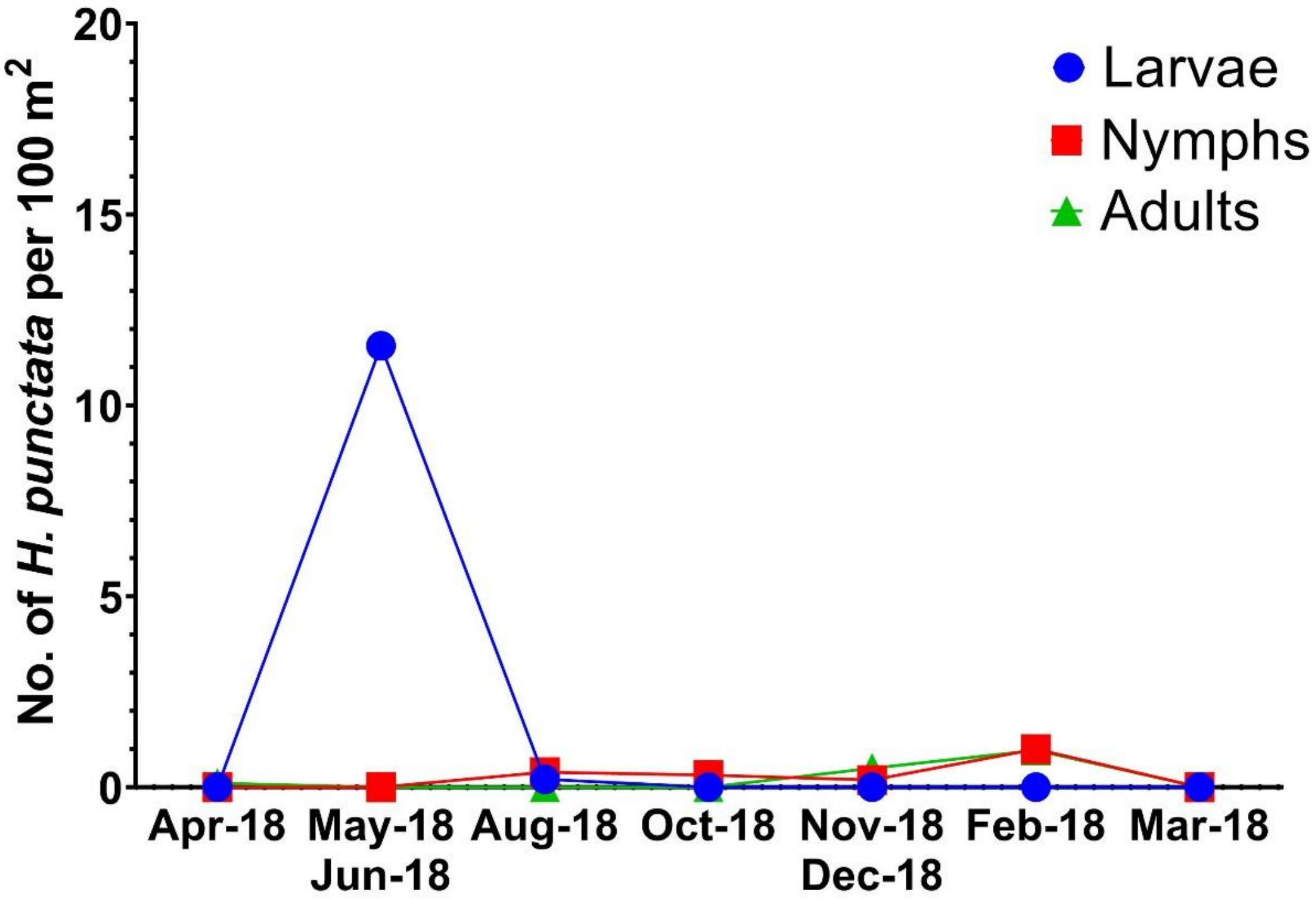

Supplementary figure 7: Abundance of *Haemaphysalis punctata* tick specimens in the seven samplings performed in the Natural Protected Area of Doñana (NPAD) and in the Natural Protected Area of Los Alcornocales (NPAA).

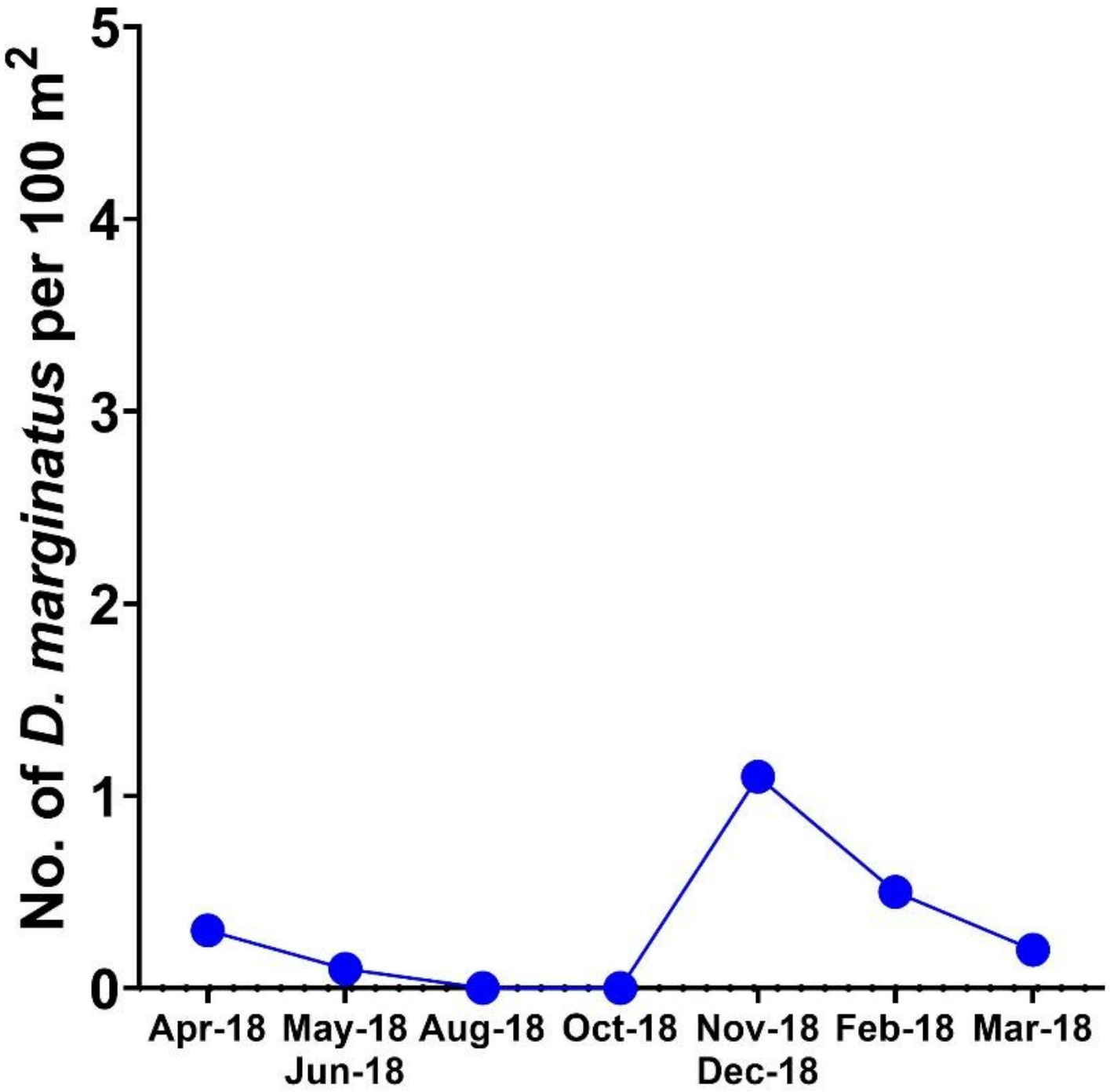

Supplementary figure 8: Abundance of adult specimens of *Dermacentor marginatus* tick specimens in the seven samplings performed in the Natural Protected Area of Doñana (NPAD) and in the Natural Protected Area of Los Alcornocales (NPAA).

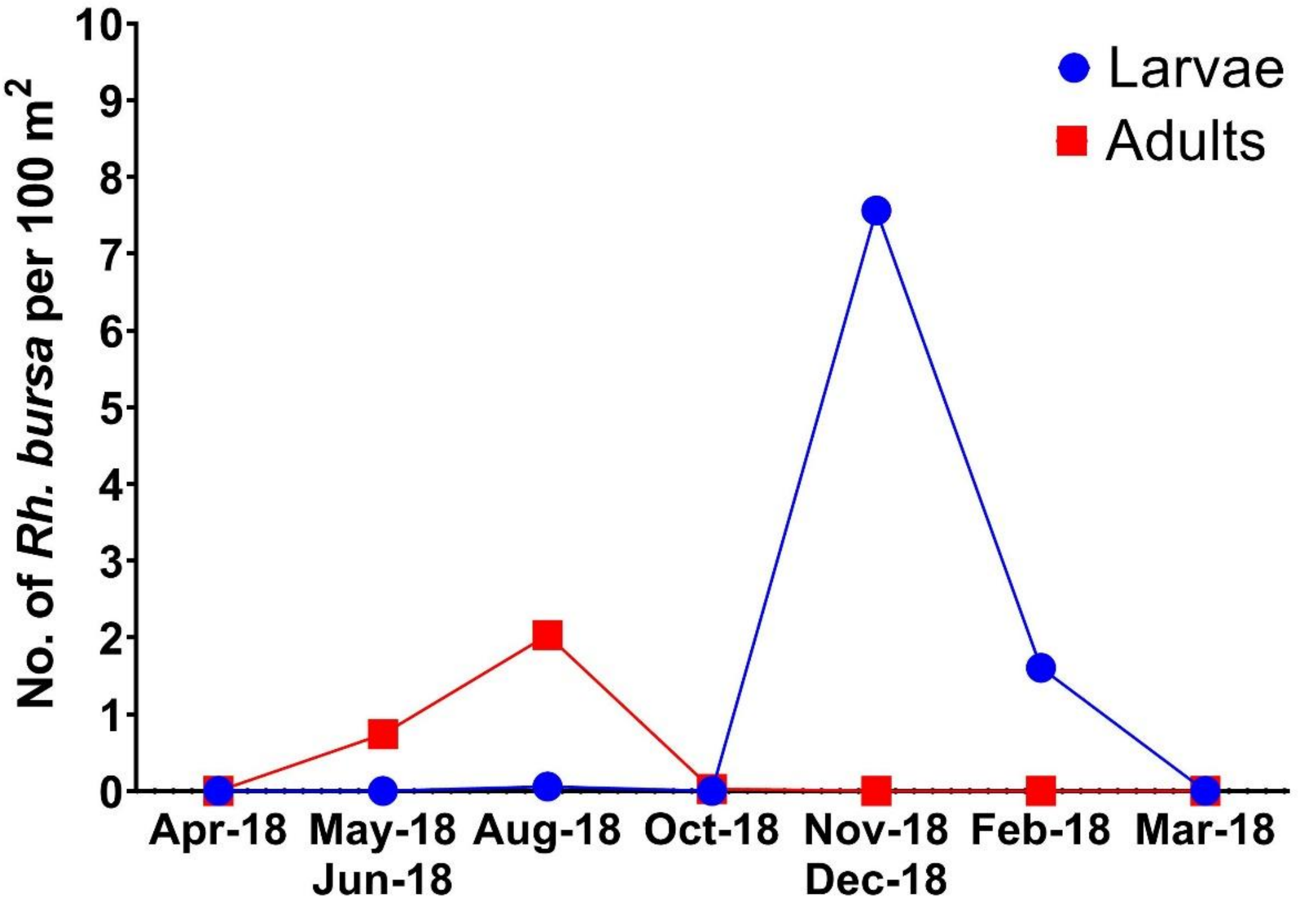

Supplementary figure 9: Abundance of *Rhipicephalus bursa* tick specimens in the seven samplings performed in the Natural Protected Area of Doñana (NPAD) and in the Natural Protected Area of Los Alcornocales (NPAA).

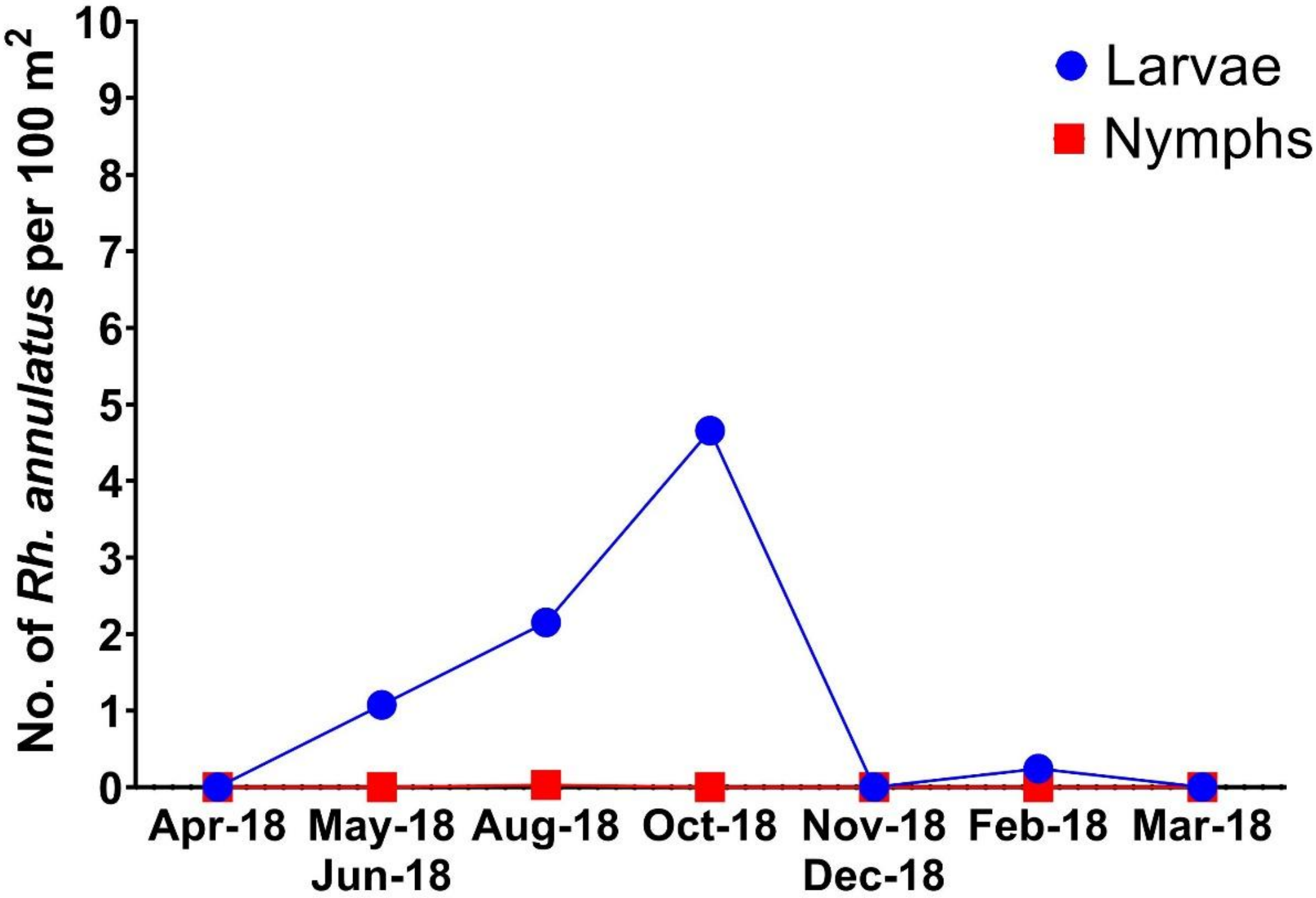

Supplementary figure 10: Abundance of *Rhipicephalus annulatus* tick specimens in the seven samplings performed in the Natural Protected Area of Doñana (NPAD) and in the Natural Protected Area of Los Alcornocales (NPAA).
